# Novel AR-12 derivatives, P12-23 and P12-34, inhibit flavivirus replication by blocking host de novo pyrimidine biosynthesis

**DOI:** 10.1038/s41426-018-0191-1

**Published:** 2018-11-21

**Authors:** Chao-Fu Yang, Balraj Gopula, Jian-Jong Liang, Jin-Kun Li, Si-Yu Chen, Yi-Ling Lee, Ching S. Chen, Yi-Ling Lin

**Affiliations:** 1 Institute of Biomedical Sciences, Academia Sinica, Taipei, 11529 Taiwan; 2 Institute of Biological Chemistry, Academia Sinica, Taipei, 11529 Taiwan; 3 Drug Development Center, China Medical University, Taichung, 40402 Taiwan; 4 Department of Medical Research, China Medical University Hospital, China Medical University, Taichung, 40447 Taiwan; 5 Genomic Research Center, Academia Sinica, Taipei, 11529 Taiwan

## Abstract

The genus *Flavivirus* contains many important pathogens, including dengue virus (DENV), Zika virus (ZIKV), and Japanese encephalitis virus (JEV). AR-12 is a celecoxib-derived anticancer agent that possesses antiviral activity against a broad range of viruses. We pharmacologically exploited this unique activity to develop additional antiviral agents, resulting in the production of the AR-12 derivatives P12-23 and P12-34. At nanomolar concentrations, these compounds were effective in suppressing DENV, ZIKV and JEV replication, exhibiting 10-fold improvements in the efficacy and selectivity indices as compared to AR-12. Regarding the mode of antiviral action, P12-23 and P12-34 inhibited viral RNA replication but had no effect on viral binding, entry or translation. Moreover, these AR-12 derivatives co-localized with mitochondrial markers, and their antiviral activity was lost in mitochondria-depleted cells. Interestingly, exogenous uridine or orotate, the latter being a metabolite of the mitochondrial enzyme dihydroorotate dehydrogenase (DHODH), abolished the antiviral activity of AR-12 and its derivatives. As DHODH is a key enzyme in the de novo pyrimidine biosynthesis pathway, these AR-12 derivatives may act by targeting pyrimidine biosynthesis in host cells to inhibit viral replication. Importantly, treatment with P12-34 significantly improved the survival of mice that were subcutaneously challenged with DENV. Thus, P12-34 may warrant further evaluation as a therapeutic to control flaviviral outbreaks.

## Introduction

*Flavivirus* is a large genus of single-stranded positive-sense RNA viruses, many of which are pathogenic and pose serious threats to global public health, such as dengue virus (DENV), Zika virus (ZIKV), Japanese encephalitis virus (JEV), yellow fever virus and West Nile virus. DENV infects humans via mosquito bites and can cause dengue fever (DF), dengue hemorrhagic fever (DHF), and severe dengue shock syndrome (DSS). Hundreds of millions of dengue infections occur annually and lead to approximately 500,000 cases of DHF/DSS and 22,000 deaths worldwide each year^1^. DENV includes four serotypes, and secondary infections caused by other viral serotypes can induce a higher risk of DHF/DSS, making vaccine development difficult^2^. ZIKV is an emerging pathogen that has caused outbreaks in 69 countries/territories, with there being evidence of ZIKV transmission since January 2015 (World Health Organization 2017 situation report). ZIKV-related microcephaly, which leads to physical and intellectual disabilities in newborns, has received global attention recently and is an unmet medical need^3,4^.

Despite rapid advances in new drug development, no anti-flavivirus drug is currently in clinical use^5^. Although some agents have been reported to inhibit flavivirus replication by targeting viral proteins such as NS3 and NS5^6^, drug resistance-associated mutations in these RNA viruses represent an urgent issue that needs to be resolved. Because viruses depend on host cells to complete their life cycles, many cellular processes, such as RNA synthesis, are essential for viral replication. From a mechanistic perspective, interfering in these cellular biological processes may be a promising strategy to develop broad-spectrum antiviral drugs that do not elicit viral resistance.

AR-12 (aka, OSU-03012) is a celecoxib derivative that was originally designed as an anticancer agent. AR-12 suppresses solid tumors and lymphoma proliferation^7,8^, and a phase I clinical study of AR-12 has been conducted (ClinicalTrials.gov no.: NCT00978523). AR-12 also exhibited antimicrobial activities against the intracellular bacteria *Salmonella* and *Francisella*^9,10^, the fungal pathogen *Candida*^11^, and the parasite *Leishmania*^12^. Furthermore, AR-12 was effective in blocking a wide-range of human pathogenic viruses, including Ebola virus, Lassa virus, influenza virus, human immunodeficiency virus and DENV^13–15^. This broad spectrum of antimicrobial activity suggests that AR-12 may target host cellular processes that are functionally conserved and essential to microbial infections. Several factors/pathways, including kinase activity, the unfolded protein response, autophagy, and acetyl CoA synthetase, have been proposed to underlie the antimicrobial effects of AR-12^9,11,13,14^. However, the detailed mechanism by which AR-12 suppresses these pathogenic microorganisms remains elusive.

**Fig. 1:**
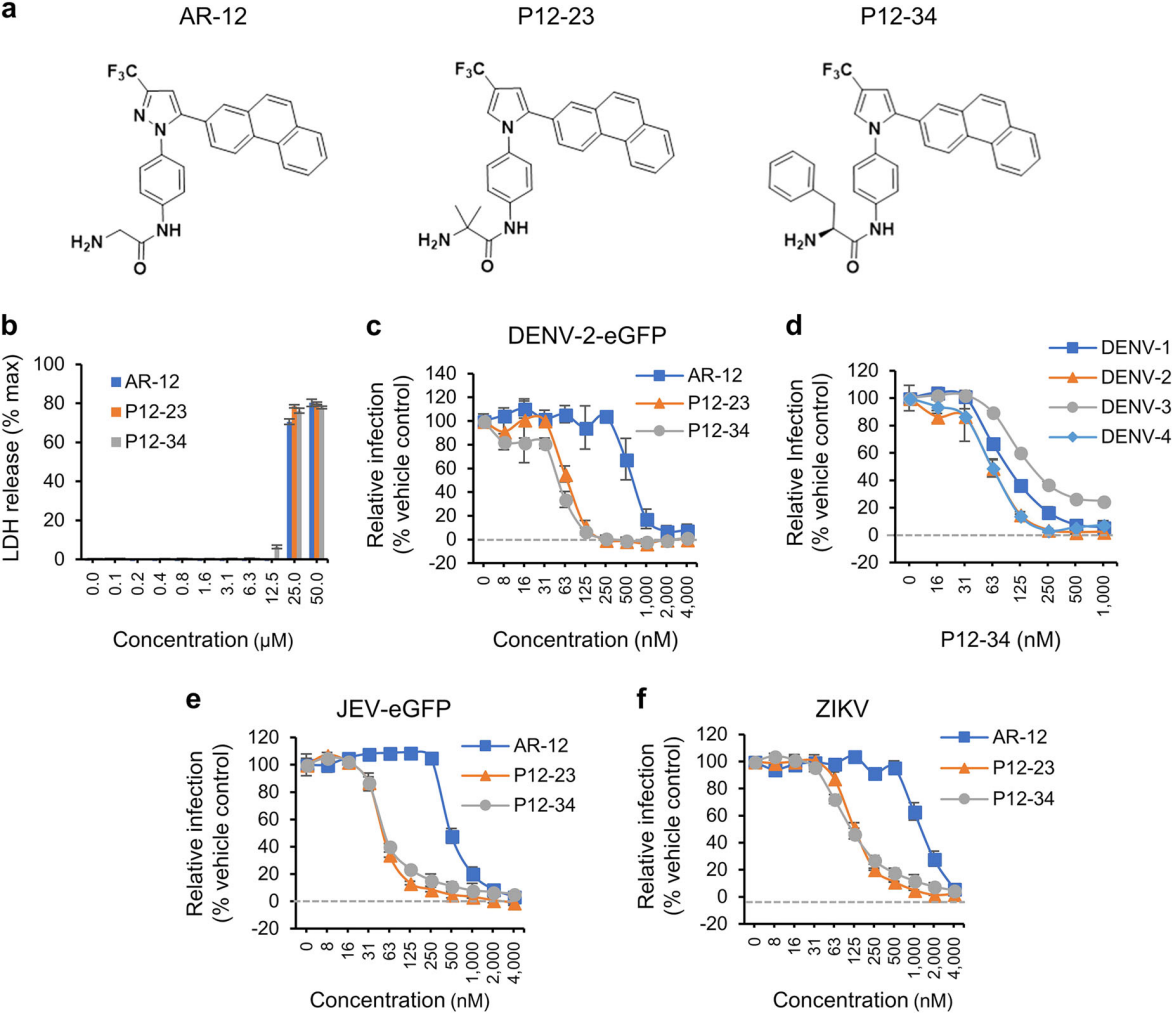
Antiviral activities of AR-12 and its derivatives, P12-23 and P12-34. **a** The structures of AR-12, P12-23, and P12-34. **b** A549 cells treated with the indicated compounds for 24 h were processed for the LDH release assay. The total amount of LDH was measured after treatment with Triton X-100 (max). **c**–**f** A549 cells treated with AR-12, P12-23, and P12-34 were infected with DENV-2-eGFP (MOI 5), DENV-1~4 (MOI 5), JEV-eGFP (MOI 1), or ZIKV (MOI 0.5) for 24 h. The protein expression of eGFP (DENV-2-eGFP and JEV-eGFP) and viral NS3 (DENV-1~4 and ZIKV) was measured using a high-content image analysis system. Data are reported as the means ± SD (*n* = 3)

To improve the antiviral potency of AR-12, we made structural modifications of the AR-12 scaffold to generate a series of derivatives that were tested for their antiviral activities against three flaviviruses, DENV, JEV and ZIKV. Among the AR-12 derivatives, P12-23 and P12-34 (structures shown in Fig. 1a) were the best antiviral agents, exhibiting substantially higher efficacy and selectivity indices against these flaviviruses compared to AR-12. We obtained evidence that mitochondria are required for their antiviral activity by inhibiting dihydroorotate dehydrogenase (DHODH), a mitochondrial enzyme involved in de novo pyrimidine biosynthesis. Moreover, P12-34 treatment protected mice against lethal challenge with DENV by a route mimicking mosquito bite, suggesting the translational potential of AR-12 derivatives in anti-flaviviral therapy.

## Results

### P12-23 and P12-34, two structurally optimized AR-12 derivatives, exhibit high antiviral efficacy, and selectivity indices against flaviviruses

To enhance the antiviral activity of AR-12, we made structural modifications of AR-12 via a two-step strategy. First, we replaced the core AR-12 structure, pyrazole, with a pyrrole ring, then substituted the terminal glycine moiety with different amino acids to generate a series of derivatives for testing. These AR-12 derivatives were evaluated for their antiviral activities using a high-content image analysis system with a DENV reporter virus, DENV-2-eGFP. Among the various derivatives examined, P12-23 and P12-34 exhibited highly improved antiviral activities and were selected as the best compounds for further studies (Fig. 1a).

Similar to AR-12, P12-23 and P12-34 were noncytotoxic to human A549 cells at concentrations of ≤12.5 µM (Fig. 1b), with 50% cytotoxic concentration (CC_50_) values of approximately 20 µM for these three compounds (Table 1). However, P12-23 and P12-34 were an order-of-magnitude more potent than AR-12 in blocking DENV-2-eGFP infection (Fig. 1c). The mean 50% inhibitory concentration (IC_50_) values for AR-12, P12-23, and P12-34 against the DENV-2 reporter virus were 660.5, 69.7 and 52.1 nM, respectively (Table 1). Accordingly, the selectivity indices (SI = CC_50_/IC_50_) were determined to be 33, 297, and 388 for AR-12, P12-23, and P12-34, respectively. Moreover, P12-34 was also effective against all four serotypes of wild-type DENV (DENV1-4), exhibiting similar IC_50_ that ranged from 62 to 98 nM (Fig. 1d and Table 1). AR-12 and its derivatives also blocked the replication of the flaviviruses JEV (Fig. 1e) and ZIKV (Fig. 1f), with P12-23 and P12-34 exhibiting a ~10-fold greater relative potency than AR-12 (Table 1). The antiviral effects of AR-12 and its derivatives were independent of the cell lines used, as similar results were obtained for human microglial CHME3 cells, DC-SIGN-expressing human monocytic THP-1 cells, baby hamster kidney BHK-21 cells and African green monkey kidney Vero cells (Supplementary Fig. S1). Overall, our data indicate that structural modifications of AR-12 generated two potent derivatives, P12-23 and P12-34, which exhibit broad-spectrum anti-flaviviral activities in multiple cell lines.

**Table 1:** Inhibitory concentrations and cytotoxic concentrations of AR-12 and its derivatives in A549 cells

Virus	Compound	CC_50_ (µM)	IC_50_ (nM)	SI (CC_50_/IC_50_)
DENV-2-eGFP	AR-12	21.4 ± 0.2	660.5 ± 130	33.2 ± 5.9
	P12-23	20.5 ± 0.1	69.7 ± 8.9	296.7 ± 35.1
	P12-34	20.3 ± 0.2	52.1 ± 2	387.9 ± 14.7
JEV-eGFP	AR-12	21.4 ± 0.2	509.9 ± 52.4	42.3 ± 4.1
	P12-23	20.5 ± 0.1	53.2 ± 1.6	385.5 ± 11.8
	P12-34	20.3 ± 0.2	56.1 ± 0.5	360.3 ± 3.1
ZIKV	AR-12	21.4 ± 0.2	1373 ± 176.6	15.8 ± 2
	P12-23	20.5 ± 0.1	130.3 ± 11.7	158 ± 13.6
	P12-34	20.3 ± 0.2	118.6 ± 11.7	171.4 ± 16.1
DENV-1	P12-34	20.3 ± 0.2	98 ± 1.7	206.3 ± 3.5
DENV-2	P12-34	20.3 ± 0.2	62.2 ± 4.9	326.5 ± 26.4
DENV-3	P12-34	20.3 ± 0.2	90.6 ± 3.4	223.4 ± 8.6
DENV-4	P12-34	20.3 ± 0.2	63.9 ± 5.1	317.8 ± 24.9

*CC*_*50*_ 50% cytotoxic concentration, *IC*_*50*_ 50% inhibitory concentration, *SI* selectivity index = CC_50_/IC_50_

### P12-23 and P12-34 suppress viral RNA replication

**Fig. 2:**
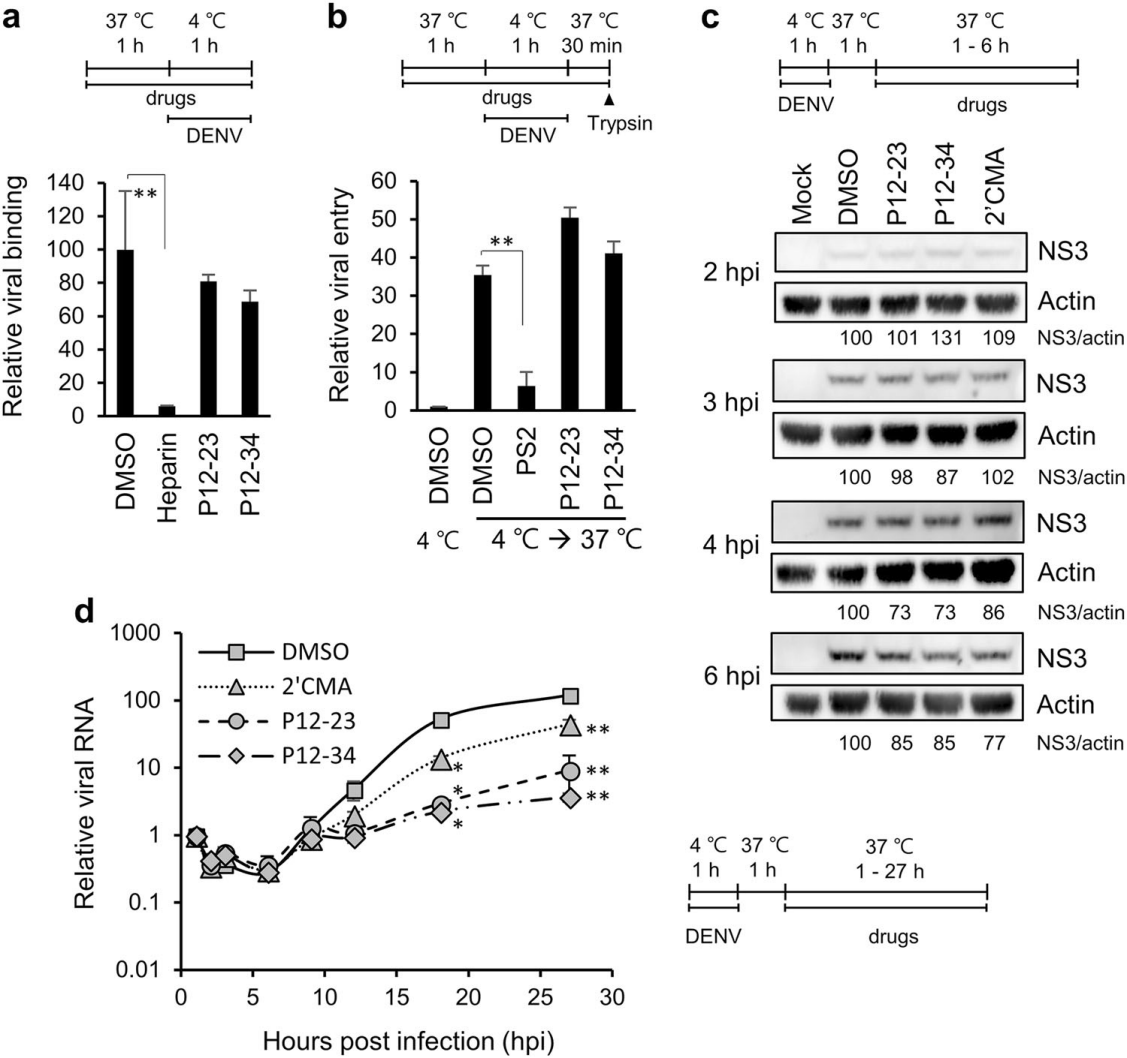
P12-23 and P12-34 inhibit dengue virus RNA replication. **a** For the viral binding assay, A549 cells and DENV-2 pretreated with P12-23, P12-34 or heparin were mixed and incubated at 4 ℃ for 1 h. Viral RNA was quantified via RT-qPCR to determine viral binding levels. **b** For the viral entry assay, A549 cells pretreated with P12-23, P12-34 or Pitstop^®^2 (PS2) were absorbed with DENV-2 at 4 ℃ for 1 h, then were shifted to 37 ℃ for 30 min. E protein-positive cells were quantified by flow cytometry after removing cell surface-associated viruses. **c** For the translation assay, DENV-2-infected A549 cells were treated with P12-23, P12-34 or 2′-C-methyladenosine (2′CMA) at 1 hpi. At the indicated times, cell lysates were harvested for western blot analysis. **d** For the viral RNA replication assay, DENV-2-infected A549 cells were treated with P12-23, P12-34 or 2′CMA at 1 hpi. Intracellular viral RNA levels were measured via RT-qPCR, and significance was determined by comparisons to the solvent control (DMSO). Data are reported as the means ± SD. *p < 0.05; **p < 0.01 by two-tailed Student’s *t*-test (*n* = 3)

To elucidate the mode of antiviral action for P12-23 and P12-34, we compared the effects of these compounds to various pharmacological inhibitors on different steps of the DENV life cycle in A549 cells. These inhibitors included the DENV binding inhibitor, heparin^16^, the clathrin-mediated endocytosis/viral entry inhibitor Pitstop®2 (PS2)^17,18^, and the NS5/viral RNA replication inhibitor 2′-C-methyladenosine (2′CMA)^19^. Compared to heparin and PS2, P12-23 and P12-34 had no suppressive effect on viral binding and entry (Figs. 2a, b). For viral translation, we monitored DENV NS3 protein expression at an early stage of infection. Similar to 2′CMA, P12-23, and P12-34 did not reduce NS3 levels until 4~6 h post-infection (hpi) (Fig. 2c), suggesting that they did not target the first round of viral translation. We further measured intracellular viral RNA levels and showed that P12-23 and P12-34 were more potent than 2′CMA in suppressing viral RNA replication (Fig. 2d). Together, these data suggest that P12-23 and P12-34 likely block DENV RNA replication and not viral binding, entry and protein translation.

### Dependence of the antiviral activity of P12-34 on mitochondria

To elucidate the antiviral mechanism of AR-12 derivatives, we first tested the reported antimicrobial targets of AR-12, such as endoplasmic reticulum (ER) stress, autophagy and GRP78 expression. Unlike the positive controls tunicamycin (TM) and thapsigargin (TG), P12-23 did not significantly induce ER stress as measured by GRP78 expression and eIF2α phosphorylation, or autophagy measured by LC3B form II formation (Supplementary Fig. S2). The reduced GRP78 expression noted in the P12-23-treated DENV-infected cells likely resulted from the suppressed viral replication of P12-23. However, the ER-stress suppressor 4-phenylbutyric acid slightly reduced the antiviral activity of P12-23, suggesting that ER stress may partially contribute to the noted antiviral effect but may not be the major antiviral mechanism.

**Fig. 3:**
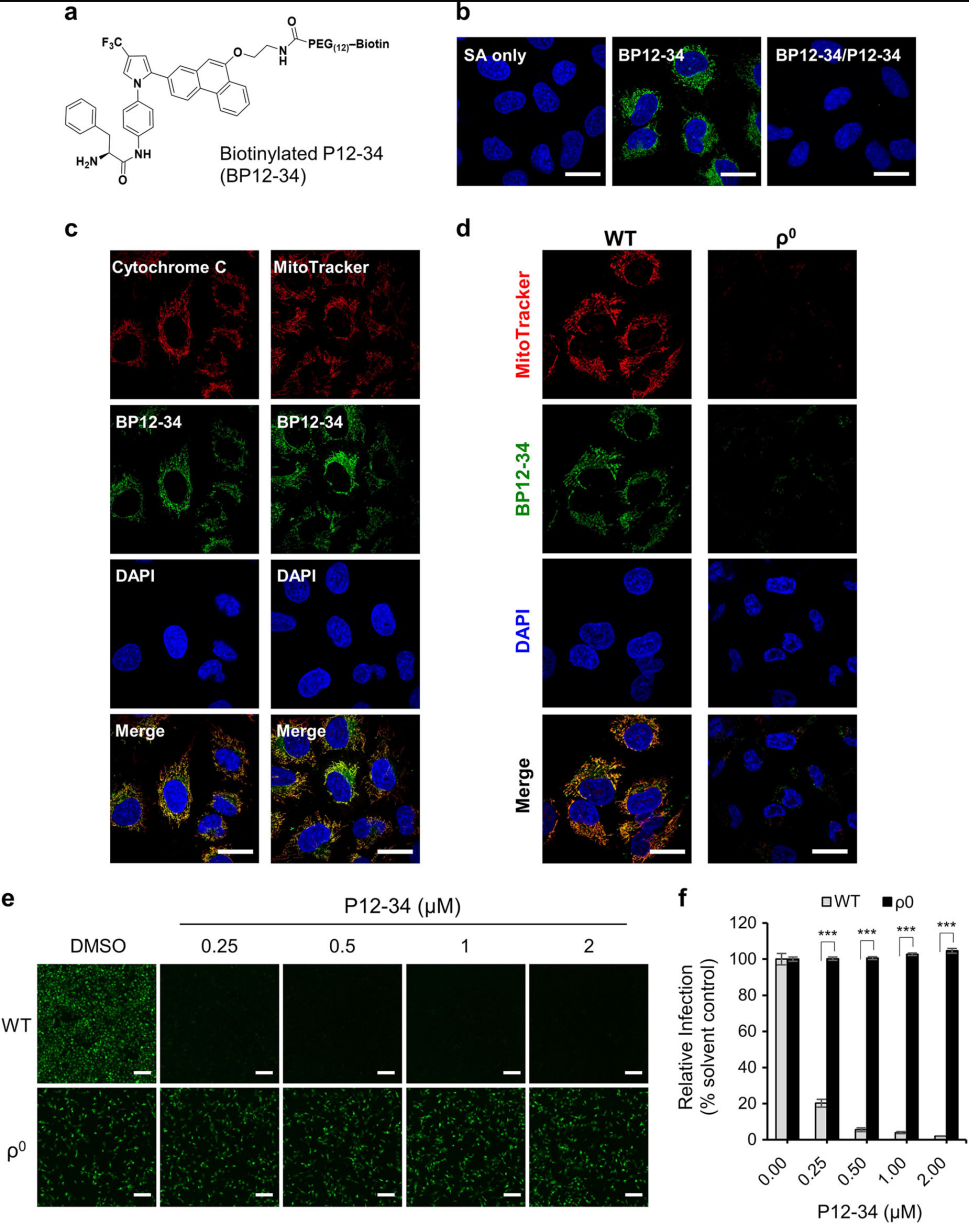
Mitochondria are required for the antiviral activity of P12-34. **a** Structure of biotinylated P12-34 (BP12-34). **b** A549 cells treated with BP12-34 were stained with fluorescent streptavidin (SA). In the BP12-34/P12-34 group, cells were pretreated with P12-34 prior to BP12-34 staining. **c** Mitochondria were stained with an anti-cytochrome c antibody and MitoTracker prior to BP12-34 staining. **d** Wild-type (WT) and mitochondria-depleted (ρ^0^) A549 cells were stained with MitoTracker and BP12-34. Nuclei were stained with DAPI. Scale bar = 20 µM. **e**, **f** WT and ρ^0^ A549 cells treated with P12-34 were infected with DENV-2-eGFP (MOI 5) for 24 h. Scale bar = 200 µM. eGFP-positive cells were measured using a high-content image analysis system and quantified using the DMSO solvent control to determine the relative viral infection. Data are reported as the means ± SEM. ***p < 0.0001 by two-tailed Student’s *t*-test (*n* = 3)

To investigate the antiviral mechanism of the AR-12 derivatives, we synthesized a biotinylated derivative of P12-34 (BP12-34) (Fig. 3a) to analyze its intracellular distribution. A549 cells were treated with BP12-34 followed by fluorescent streptavidin. Fluorescent imaging of these cells revealed a cytoplasmic filamentary staining pattern that was not seen in cells treated with streptavidin alone and could be blocked by P12-34 (Fig. 3b). To identify these filamentary structures, we costained BP12-34-treated cells with different organelle-specific markers. The BP12-34 signals overlapped with those of mitochondrial markers, including cytochrome c and MitoTracker (Fig. 3c), but not with those of the autophagosome, ER or Golgi markers (Supplementary Fig. S3). Furthermore, we established mitochondria-depleted (ρ^0^) A549 cells by continuously exposing cells to ethidium bromide for more than 3 months. Compared to the wild-type (WT) A549 cells, the staining signal of BP12-34 was lost in the ρ^0^ cells (Fig. 3d), indicating that mitochondria were targeted by P12-34. Furthermore, the anti-DENV activity of P12-34 was lost in ρ^0^ A549 cells (Figs. 3e, f), confirming the involvement of mitochondria in the antiviral effect of P12-34. It is interesting that depletion of mitochondria did not suppress DENV replication, suggesting the existence of a compensatory mechanism from other cellular compartments to support viral RNA synthesis.

### P12-23 and P12-34 antiviral effects are mediated by the inhibition of mitochondrial pyrimidine biosynthesis in host cells

**Fig. 4:**
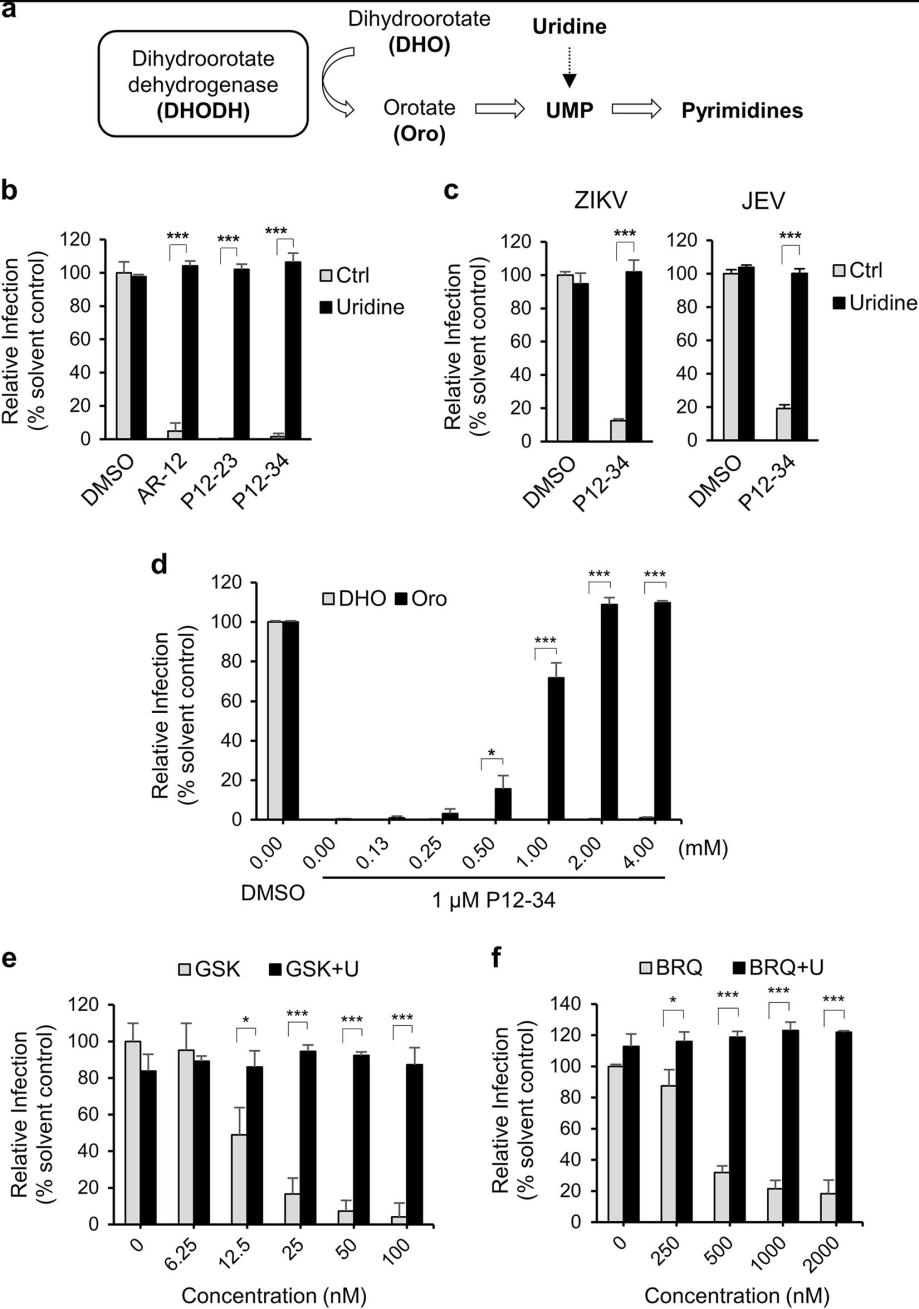
P12-23 and P12-34 block viral replication by interfering with host pyrimidine biosynthesis. **a** Dihydroorotate dehydrogenase (DHODH) converts dihydroorotate (DHO) to orotate (Oro) for pyrimidine biosynthesis. **b** A549 cells infected with DENV-2-eGFP (MOI 5) were treated with P12-23 (1 µM), P12-34 (1 µM) or AR-12 (5 µM) in medium with or without uridine (50 µg/ml). **c** A549 cells infected with ZIKV (MOI 0.5) or JEV-eGFP (MOI 1) were treated with P12-34 (1 µM) with or without supplemented uridine (50 µg/ml). **d** A549 cells infected with DENV-2-eGFP (MOI 5) were treated with P12-34 (1 µM) in the presence of DHO or Oro. **e** A549 cells infected with DENV-2-eGFP (MOI 5) were treated with GSK983 (GSK) or brequinar (BRQ) in the presence or absence of 50 µg/ml uridine (U). eGFP or NS3 (ZIKV) expression was measured using a high-content image analysis system at 24 hpi. Data are reported as the means ± SD. *p < 0.05; ***p < 0.0001 by two-tailed Student’s *t*-test (*n* = 3)

Because AR-12 and its derivatives could block viral RNA replication, we hypothesized that these agents may target mitochondrial pyrimidine biosynthesis, particularly that of uridine monophosphate (UMP). To test this hypothesis, we performed an anti-DENV assay using uridine-supplemented versus regular culture medium. In this assay, because uridine can be converted to UMP via the salvage pathway, cells can bypass the need for de novo pyrimidine biosynthesis in mitochondria^20^ (Fig. 4a). Interestingly, the uridine supplement abolished the anti-DENV activity of AR-12 and its derivatives (Fig. 4b). Similarly, this effect of uridine was also noted in the P12-34-mediated suppression of JEV and ZIKV replication (Fig. 4c), demonstrating that this effect was not DENV-specific.

To identify the mitochondrial target upon which these antiviral agents act to inhibit UMP production, we turned our attention to DHODH, which is involved in the de novo pyrimidine biosynthesis pathway in mitochondria. Specifically, DHODH oxidizes dihydroorotate (DHO) to form orotate (Oro), which is then converted to UMP (Fig. 4a)^21^. Based on this premise, we assessed the antiviral effect of P12-34 in cell medium supplemented with the substrate (DHO) or product (Oro) of DHODH. Consistent with our premise, the addition of Oro but not DHO could block the suppressive effect of P12-34 on DENV replication (Fig. 4d). In addition, two DHODH inhibitors, GSK983 and brequinar^22,23^, reduced DENV replication in a dose-dependent manner, which could be reversed by an exogenous uridine treatment (Figs. 4e, f), similar to that noted for P12-23 and P12-34. To address whether AR-12 derivatives directly or indirectly target DHODH, we used BP12-34 to pull down the associated proteins. P12-34 did not appear to interact with DHODH (Supplementary Fig. S4). Instead, P12-34 was observed to bind cytochrome bc1 complex in a dose-dependent manner. Cytochrome bc1 complex is present in mitochondria and controls the cycling of ubiquinone, a crucial coenzyme that is required for DHODH to accept electrons during the oxidation of DHO to Oro. The inhibition of cytochrome bc1 complex has been reported to regulate DHODH function^24,25^. Thus, AR-12 and its derivatives may interfere with DHODH activity by targeting cytochrome bc1 complex.

Since DHODH inhibitors have be observed to suppress viral growth through innate immunity^26,27^, we tested whether AR-12 and its derivatives possess the ability to amplify the cellular innate immune response. Similar to the DHODH inhibitor brequinar, P12-34 enhanced the innate immune responses triggered by short 5’-triphosphate RNA molecules (ssRNA) as measured by the interferon (IFN)-stimulated response element (ISRE) reporter (Supplementary Fig. S5a) and the IFN-stimulated expression of downstream genes, such as IFN-induced protein with tetratricopeptide repeats 1 (IFIT1) and IFN-regulatory factor 1 (IRF-1) (Supplementary Fig. S5b). Thus, our results suggest that similar to other DHODH inhibitors, AR-12 and its derivatives can also amplify innate immunity, which then may contribute to their antiviral activities.

### P12-34 protects mice against lethal DENV challenge

**Fig. 5:**
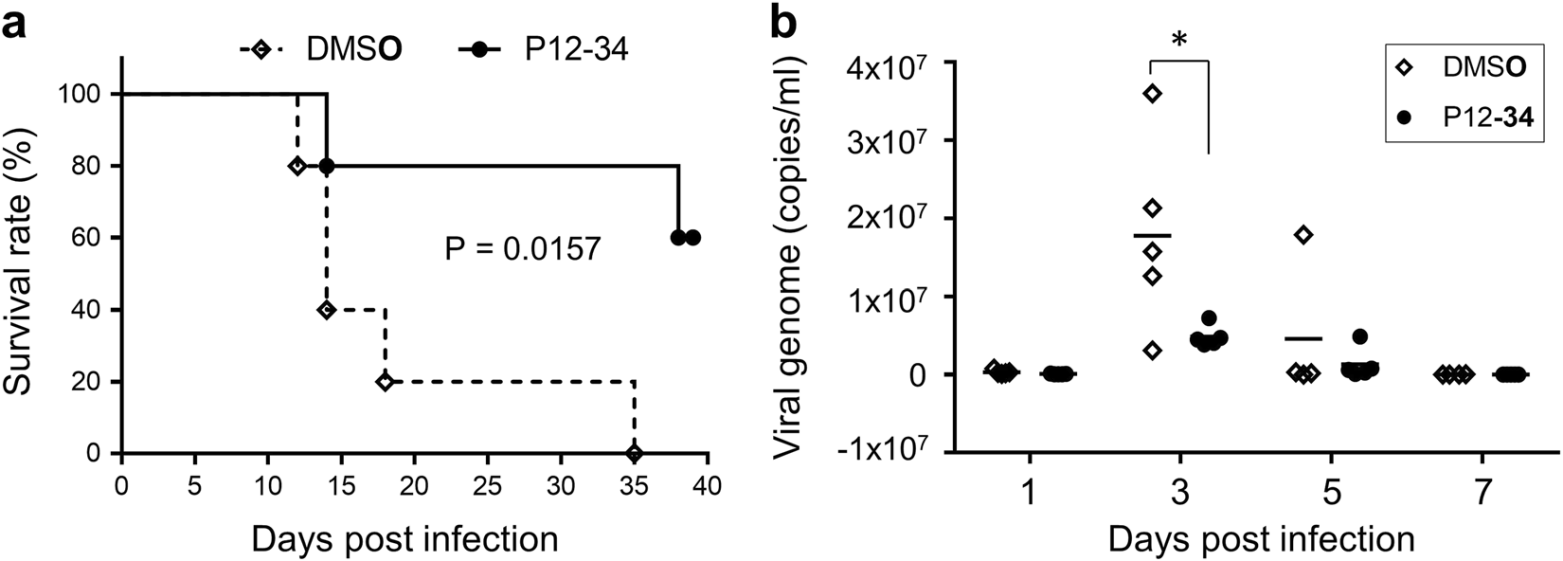
P12-34 protects mice against lethal DENV challenge. Stat1^−/−^ mice subcutaneously challenged with a lethal dose of DENV-2 were intraperitoneally treated with P12-34 (2.5 mg/kg per day) or vehicle control (DMSO) for the first 6 days of infection. **a** Survival of mice with or without P12-34 treatment. The P value was determined using a Log-rank (Mantel-Cox) test (*n* = 5). **b** The viral loads in sera quantified by RT-qPCR are shown as the mean (bar) and as individual data. *p < 0.05, determined by two-tailed Student’s *t*-test (*n* = 5)

To evaluate the in vivo antiviral efficacy of P12-34, we used STAT1-deficient mice lacking the interferon response as a DENV challenge model^28,29^. Stat1^−/−^ mice were subcutaneously injected with a mouse-adapted DENV strain and randomly divided into two groups (*n* = 5 for each group) that received daily intraperitoneal injections of P12-34 (2.5 mg/kg) or vehicle for the first 6 days of infection. All mice in the vehicle control group died within 35 days post-infection (dpi), with a median survival time of 14 days (Fig. 5a). In contrast, 60% of the P12-34-treated mice were alive at 39 dpi and did not exhibit noticeable symptoms (Fig. 5a). Furthermore, the viral genome copy numbers were significantly reduced in the P12-34 group relative to the control, especially at 3 dpi (Fig. 5b). In addition, similar to a previously reported DHODH inhibitor^30^, P12-34 did not protect mice against a DENV challenge when the virus was injected intraperitoneally (data not shown), probably because of the presence of high amounts of uridine from liver limiting the antiviral effect of P12-34^31^.

## Discussion

Many important pathogenic flaviviruses are transmitted by insect vectors in tropical and subtropical areas. Global warming and international travel facilitate the spread of flaviviruses, and emerging/reemerging outbreaks have occurred, such as the ZIKV outbreak in 2015. The development of broad-spectrum antiviral drugs might be a viable strategy to control future flaviviral epidemics. Since there is a great demand for nucleic acids during the course of viral replication, reducing the cellular nucleotide pool may have a broad-spectrum antiviral effect^32,33^. Ribavirin, which inhibits inosine monophosphate dehydrogenase to decrease intracellular purine levels, exhibits broad-spectrum antiviral activity and has been used in anti-hepatitis C viral therapy^33,34^. In this study, we developed two novel compounds, P12-23 and P12-34, for use against several flaviviruses based on the scaffold of AR-12, a celecoxib derivative designed for anticancer therapy. We showed that P12-23 and P12-34 inhibited mitochondrial de novo pyrimidine biosynthesis to suppress viral RNA replication.

Pyrimidine nucleotides play crucial roles in cellular metabolism, including serving as RNA and DNA building blocks and as components of CDP-diacylglycerol phosphoglyceride for cell membranes and UDP-sugars for protein glycosylation and glycogen synthesis^35^. Thus, pyrimidines are involved in RNA and DNA synthesis, cell membrane assembly and protein glycosylation. Decreasing the pyrimidine pool by blocking DHODH can induce autophagy^36^, suppress the phosphoinositide-dependent kinase 1/Akt pathway^37^, induce ER stress, and activate the UPR^38^. The ability of AR-12 to regulate these cellular processes underlies its anticancer and antiviral effects^14,39–41^. Our data suggest that AR-12 and its derivatives interfere with the activity of DHODH, probably by targeting cytochrome bc1 complex in mitochondria, which leads to metabolic stress in drug-treated cells. Mechanistically, AR-12-regulated cellular pathways may be the downstream outcome of pyrimidine biosynthesis suppression.

DHODH has been extensively explored as a potential drug target in rheumatology, oncology and infectious diseases studies^21^. DHODH inhibitors, including the US Food and Drug Association-approved drug brequinar, exhibit broad-spectrum antiviral activities in cultured cells^30,32,42–44^. However, DHODH inhibitors have failed to show promising antiviral effects in animal models^30,42,44^. The discrepancy between the in vitro and in vivo efficacies of these drugs may be due to the coexistence of two pyrimidine biosynthesis pathways, namely, the de novo and salvage pathways^35^. Plasma uridine can regulate pyrimidine biosynthesis via the salvage pathway and decrease the dependency of cells on de novo-derived pyrimidines^45^. Consequently, a high concentration of uridine in the liver^31^ may hinder the antiviral effect of AR-12 derivatives. Therefore, we assessed both subcutaneous and intraperitoneal routes of DENV challenge in our in vivo antiviral assays. Interestingly, P12-34 protected mice against DENV infection with viral challenge by the subcutaneous but not intraperitoneal route (Fig. 5 and data not shown). The antiviral effect of P12-34 observed by the subcutaneous challenge is encouraging, because it mimics the mosquito bite transmission of flaviviruses in humans.

In summary, in this study, we synthesized two novel AR-12 derivatives with potent antiviral activity against several flaviviruses. Equally important, we identified a new mode of action for AR-12 and its derivatives, which occurs by interfering with the mitochondrial enzyme DHODH. DHODH catalyzes the oxidation of DHO to Oro in a conserved enzymatic reaction associated with the de novo pyrimidine biosynthesis pathway. The inhibition of pyrimidine biosynthesis is a strategy used to treat various diseases, such as cancer, immunological disorders and infections, and the development of DHODH inhibitors has been under continuous investigation^21^. The addition of AR-12 and its derivatives to the growing list of DHODH inhibitors opens up new avenues of drug development. Strategies such as the control of plasma uridine or the suppression of pyrimidine salvage biosynthesis could be explored to boost the in vivo effect of AR-12 and its derivatives. Our finding that broad-spectrum anti-flaviviral agents target the cellular pyrimidine pool may prove useful in strategies to control future flaviviral outbreaks.

## Materials and methods

### Cell lines, viruses, and chemicals

Human lung epithelial A549 cells (American Type Culture Collection, Manassas, USA, CCL-185) were maintained in F-12 medium (ThermoFisher, NY, USA) containing 10% fetal bovine serum. To deplete mitochondria, A549 cells were continually treated with 50 ng/ml EtBr over 3 months and maintained in F-12 medium containing 50 µg/ml uridine^20^.

DENV-1 (Hawaii), DENV-2 (PL046), DENV-3 (H087), DENV-4 (H241), and ZIKV (PRVABC59) were amplified in C6/36 cells. Viral titers were determined by plaque assay as described previously^46^. AR-12 was a kind gift from Arno Therapeutics (Flemington, NJ). Other cell lines and details regarding culture conditions, the production of reporter virus, the synthesis of P12-23, P12-34, and BP12-34, and the inhibitors used in this study are described in the Supplementary Information.

### Cytotoxicity assay

Cells were treated with compounds at the indicated concentrations for 24 h. The release of lactate dehydrogenase (LDH) was measured using a Cytotoxicity Detection Kit (Roche, Basel, Switzerland) after removing cell debris. Cells treated with 1% Triton X-100 were used to determine the maximal LDH release.

### High-content image analysis system

Approximately 50% of confluent cells seeded in 96-well plates were infected with viruses in the presence of drugs or DMSO as a solvent control. At 24 h post-infection (hpi), cells were fixed with 4% paraformaldehyde at room temperature (RT) for 20 min, permeated with a 0.5% Triton X-100 buffer for 2 min, and blocked with 3% BSA for 30 min. Nuclei were stained with DAPI (Sigma) at RT for 20 min. Viral NS3 was stained with a mouse anti-NS3 monoclonal antibody (mAb)^47^ followed by staining with an Alexa Fluor 488-conjugated goat anti-mouse IgG antibody (ThermoFisher) at RT for 1 h. Nine images from different fields per well were acquired and analyzed using an ImageXpress Micro XLS Widefield High-Content Analysis System (Molecular Devices, Sunnyvale, USA). To quantify viral infection, the fluorescence intensities of viral NS3 or eGFP from the reporter virus were measured using MetaXpress® (Molecular Devices, Sunnyvale, USA) and were calculated with a mock control (infection as 0%) and a DMSO solvent control (infection as 100%).

### Viral binding assay

A549 cells and DENV-2 separately pretreated with P12-23 (1 µM), P12-34 (1 µM) or heparin (100 unit/ml) at 37 ℃ for 1 h were mixed to allow for viral adsorption at a multiplicity of infection (MOI) of 5 at 4 °C for 1 h. After washing off unbound viruses, RNA was extracted using an RNeasy^®^ Mini Kit (QIAGEN, Hilden, Germany), and viral titers were quantified by real-time RT-PCR as previously described^48^ using DENV-2 primers (5′-CAATATGCTGAAACGCGAGAGAAA-3′ and 5′-AAGACATTGATGGCTTTTGA-3′) and β-actin primers (5′-TCCTGTGGCATCCACGAAACT-3′ and 5′-GAAGCATTTGCGGTGGACGAT-3′). After the RNA was normalized to the internal control in each sample, the relative level of viral RNA was determined using the DMSO solvent control.

### Viral entry assay

A549 cells pretreated with P12-23 (1 µM), P12-34 (1 µM) or Pitstop^®^2 (5 µM) at 37 ℃ for 1 h were absorbed with DENV-2 (MOI 5) at 4 °C for 1 h. After removing the unbound viruses, cells were shifted to 37 ℃ for 30 min for viral entry. The cell surface-associated viruses were then removed by treatment with trypsin-EDTA (ThermoFisher) at 37 °C for 10 min. Viral entry indicated by E protein positivity was determined using a BD FACSCalibur Flow Cytometer (BD) after immunofluorescent staining with an anti-E mAb^47^.

### Viral translation assay

A549 cells absorbed with DENV-2 (MOI 20) at 4 °C for 1 h were washed to remove unbound viruses. After incubating at 37 °C for 1 h to allow viral entry, cells were treated with P12-23 (1 µM), P12-34 (1 µM) or 2′-c-methyladenosine (20 µM). Cells were harvested at 2, 3, 4, and 6 hpi for western blot analysis as previously described^46^ with an anti-NS3 mAb^47^ and an anti-actin mAb (NB600-501; Novus Biologicals, Littleton, CO, USA). Protein expression levels were quantified using ImageJ.

### Viral RNA replication assay

Viral infection (MOI 5) and drug treatment were performed as described for the virus translation assay. Cells were harvested for real-time RT-PCR analysis after removing the cell-free viruses at 1, 2, 3, 6, 9, 12, 18, and 27 hpi. Viral RNA was quantified and normalized with actin RNA as described for the virus binding assay.

### Immunofluorescence (IF) and fluorescence staining

A549 cells were cultured to approximately 80% confluence on coverslips. For mitochondrial signals, cells were stained with 500 nM MitoTracker® Orange CMTMRos (M7510, ThermoFisher) at 37 °C for 1 h. Next, the cells were fixed, permeated and blocked as described for the high-content image analysis system for IF staining with organelle-specific primary antibodies as described in the Supplementary Information. To analyze the intracellular distribution of P12-34, cells were stained with 500 nM biotinylated P12-34 (BP12-34) at RT for 30 min and then were stained with Alexa Fluor 488-conjugated streptavidin (ThermoFisher) at RT for 20 min after fixation and permeation. In the competition assay, cells were pretreated with 50 µM P12-34 at RT for 30 min and were further stained with BP12-34 and streptavidin. Images were acquired under a Zeiss LSM700 confocal microscope.

### Animal study

Animal studies followed the guidelines of the Academia Sinica Institutional Animal Care and Use Committee. Five-week-old Stat1^−/−^ mice were subcutaneously challenged with 1 × 10^5^ plaque-forming units (PFU) of DENV-2 (mouse-adaptive NGC strain)^29^. P12-34 (2.5 mg/kg per day in 500 µl PBS containing 10% DMSO) or vehicle control was intraperitoneally injected into DENV-2-infected mice for the first 6 days. At 1, 3, 5 and 7 days post-inoculation (dpi), the viral loads in sera were determined by RT-qPCR as previously described^49^.

### Statistical analysis

Representative data from repeated experiments are presented as the means and SD (three independent samples, *n* = 3) and were analyzed by two-tailed Student’s *t*-test. Significance was determined at *p* < 0.05, and survival curves were analyzed using a Log-rank (Mantel-Cox) test (*n* = 5).

## Electronic supplementary material

Supplementary Information

Supplementary figure 1

Supplementary figure 2

Supplementary figure 3

Supplementary figure 4

Supplementary figure 5
