# Supplementary material for: Novel AR-12 derivatives, P12-23 and P12-34, inhibit flavivirus replication by blocking host de novo pyrimidine biosynthesis
Source: Emerg Microbes Infect. 2018 Nov 21;7:187. doi: 10.1038/s41426-018-0191-1 (PMC6246607; doi:10.1038/s41426-018-0191-1)
Supplement: Supplementary file 1 — Supplementary Information [file 41426_2018_191_MOESM1_ESM.docx]

**Novel AR-12 derivatives, P12-23 and P12-34, inhibit flavivirus replication by blocking host *de novo* pyrimidine biosynthesis**

**Supplementary Information**

**Cell lines, chemicals and antibodies**

Human microglial CHME3 cells and human embryonic kidney 293T (HEK 293T) (ATCC, CRL-3216) cells were cultured in Dulbecco modified Eagle medium (DMEM) (ThermoFisher) containing 10% FBS. DC-SIGN–expressing human monocytic THP-1 cells ^1^ and baby hamster kidney cells (BHK-21) (ATCC, CCL-10) were grown in RPMI medium (ThermoFisher) containing 10% and 5% FBS, respectively. African green monkey kidney Vero cells (ATCC, CCL-81) were cultured in Minimum Essential Medium (MEM) (ThermoFisher) containing 10% FBS. *Ae. albopictus* C6/36 cells (ATCC, CRL-1660) were maintained in RPMI medium (ThermoFisher) containing 10% FBS. Mammalian cells were cultured at 37 °C in a 5% CO_2_ incubator. Insect cells were cultured at 28 °C in a 5% CO_2_ incubator.

DENV binding inhibitor heparin (H3393), DHODH inhibitors brequinar (SML0113) and GSK983 (SML1824), tunicamycin (T7765), thapsigargin (T9033), 4-phenylbutyric acid (P21005), dihydroorotate (D7128) and orotate (O2750) were from Sigma (St. Louis, MO, USA). Clathrin-mediated endocytosis inhibitor Pitstop^®^2 (ab120687) was from Abcam (Cambridge, MA, USA). NS5 inhibitor 2′-c-methyladenosine (sc-283467) was from Santa Cruz Biotechnology (Dallas, TX, USA). High Capacity Magne® Streptavidin Beads (V7820) for pull down assay were from Promega (Madison, WI, USA).

Antibodies against eIF2α (#9722), phospho-eIF2α (#9721), GRP78 (#3177), and LC3B (#2775) were from Cell Signaling (Danvers, MA, USA); DHODH (GTX32560), cytochrome bc1 complex subunit 1 (UQCRC1) (GTX101896), UQCRC2 (GTX114873), and interferon-induced protein with tetratricopeptide repeats 1 (IFIT1) (GTX103452) were from GeneTex (Irvine, CA, USA); Cytochrome b was from Abcam; interferon regulatory factor 1 (IRF-1) was from Santa Cruz (sc-497). Organelle-specific primary antibodies for mitochondria: mouse anti-cytochrome c mAb (#556433; BD, Franklin Lakes, NJ, USA); endoplasmic reticulum (ER): rabbit anti-calnexin mAb (#2679, Cell Signaling); Golgi: mouse anti-GM130 mAb (#610823, BD); and autophagosome: rabbit anti-LC3B polyclonal antibody (#2775, Cell Signaling). Secondary antibodies: goat Alexa Fluor 568-conjugated anti-mouse and anti-rabbit IgG antibodies (ThermoFisher).

**Construction of reporter virus JEV-eGFP and DENV-2-eGFP**

The JEV infectious clone CMV RP-9 ^2^ was used to construct the JEV reporter virus as previously described for DENV ^3^ with modifications. In brief, nucleotides 1-4214 of the JEV genome was PCR-amplified and subcloned into pJET1.2 as the shuttle vector JEV 1-4214/pJET1.2. eGFP cDNA was inserted into this shuttle vector upstream of the JEV ATG start site by a standard oligonucleotide ligation strategy. The porcine teschovirus (PTV1) 2A sequence was inserted between the eGFP and ATG by reverse PCR with primers containing half of the PTV1 2A sequences. The JEV cyclization sequences of the shutter vector were silently mutated by single primer PCR mutagenesis. Sequences encoding the ﬁrst 34 amino acids of the JEV capsid protein and the cyclization sequences were inserted into the shuttle vector by PCR and blunt-end ligation. The ﬁnal shuttle clone containing the duplicated capsid element followed by eGFP-PTV2A, and the 5′ end of JEV genome with a mutated cyclization sequence was cloned back to the JEV infectious clone CMV RP-9 to generate CMV RP-9 C34-eGFP-2A 5′CS mut. For infectious virus production, the plasmid was transfected into BHK-21 cells with Lipofectamine 2000. Culture supernatants were collected after incubation for 4 days, then the viruses were amplified in C6/36 cells.

For DENV reporter virus, we established a DENV-2 infectious clone with the SP6 promoter (SP6-Den2). Briefly, DENV-2 PL046 RNA was extracted, underwent RT-PCR to produce five fragments containing nucleotides 1-1512, 1342-4250, 4132-6375, 4522-9368 and 6838-10723 and cloned in the polylinker of pJET1.2. The full-length cDNA clone was generated by a standard restriction enzyme and ligation strategy with pBR322 used as a vector. For constructing the DENV-2-eGFP reporter virus, the eGFP-2A sequence was PCR-amplified from the CMV RP-9 C34-eGFP-2A 5′CS mut and inserted into SP6-Den2 between the 5′ UTR and DENV-2 ATG start site. The DENV-2 cyclization sequence was silently mutated by single primer PCR mutagenesis. Sequences encoding the ﬁrst 34 amino acids of DENV-2 capsid protein and the cyclization sequence were inserted into the SP6-Den2 infectious clone by PCR and blunt-end ligation. The ﬁnal clone containing the duplicated capsid element followed by eGFP-PTV2A and the 5′ end of the DENV-2 genome with a mutated cyclization sequence was named SP6-Den2 C34-eGFP-2A 5′CS mut. For viral production, the viral RNA was in vitro transcribed from the SP6 promoter and transfected into BHK-21 cells with Lipofectamine 2000. The virus was amplified in C6/36 cells for further study.

**Interferon-stimulated response elements (ISRE) reporter assay**

Short synthetic 5′-triphosphate RNA molecules (ssRNA) was synthesized with use of the RiboMAX™ Large Scale RNA Production Kit (P1280, Promega) from control DNA template in the kit, then extracted with the RNeasy® Mini Kit (QIAGEN). About 80% confluent HEK 293T cells in 48 well-plate were co-transfected with pISRE-Luc (Stratagene, Ref 219089) (10 ng/well) and pRL-TK *Renilla* reporter plasmids (E2241, Promega) (2 ng/well), and increasing doses of ssRNA, then treated with DMSO, 1 μM brequinar, or 1 μM P12-34 for 24h. The luciferase activities were analyzed by using the Dual-Luciferase® Reporter Assay Kit (Promega) and SpectraMax L Microplate Reader (Molecular devices, San Jose, CA, USA). ISRE-Luciferase activity was firstly normalized with *Renilla*-luciferase activity, then normalized with DMSO control without ssRNA.

**Synthesis of P12-23, P12-34, and Biotinylated P12-34 (BP12-34)**

**(I) General Materials and Methods**

All commercially available reagents were used without further purification unless otherwise stated. Anhydrous tetrahydrofuran (THF) was obtained by distilling commercial reagent over CaH_2_ and anhydrous dihydrofuran (DMF) was obtained by distillation over P_2_O_5_ under reduced pressure. Silica gel for column chromatography was from Fisher Scientific (230–400 mesh). Routine ^1^H and ^13^C nuclear magnetic resonance (NMR) spectra were recorded on a Bruker instrument (DPX 300, Ascend 400, AV400 or AVII 500). Samples were dissolved in deuterated chloroform (CDCl_3_) or dimethyl sulfoxide (DMSOd_6_) and tetramethylsilane (TMS) was a reference. Electrospray ionization mass spectrometry analyses were performed with a Bruker maXis 4G mass spectrometer. All compounds for bioassay were identified with ^1^H NMR, ^13^C NMR and HRMS, with purities confirmed to be higher than 95%.

1. **General synthetic procedure for synthesis of P12-23 and P12-34 compounds:**

**Synthesis of 2-(1,1-Diethoxyethyl)phenanthrene (1a):** *Step a*. NBS (0.121 g, 0.68 mmol) was added to a solution of 2-acetyl phenanthrene (5.0 g, 22.69 mmol) and (EtO)_3_CH (6.72 g, 45.39 mmol) in absolute ethanol (50 mL), and the resulting solution was stirred at room temperature for 5 to 6 h. After completion of the reaction monitored by thin-layer chromatography (TLC), a cold aqueous solution of NaOH (10%, 15 mL) was added and the mixture was extracted with Et_2_O (3 × 50 mL). The organic extracts were washed with water (3 ×25 mL) and dried over anhydrous Na_2_SO_4_. Evaporation of the solvent under reduced pressure gave crude product. Further purification was preceded by triethylamine-coated flash column chromatography to give 2-(1,1-diethoxyethyl)phenanthrene **(1a)** (5.4 g) 82% yield. ^1^H NMR (400 MHz, CDCl_3_) δ: 8.67 (t, *J* = 9.0 Hz, 2H), 8.08 (s, 1H), 7.88 (d, *J* = 8.0 Hz, 1H), 7.80 ~ 7.73 (m, 3H), 7.66 ~ 7.63 (m, 1H), 7.59 ~ 7.56 (m, 1H), 3.59 ~ 3.53 (m, 2H), 3.49 ~ 3.41 (m, 2H), 1.68 (s, 3H), 1.29 ~ 1.22 (m, 6H).

**4-Ethoxy-1,1,1-trifluoro-4-(phenanthren-2-yl)but-3-en-2-one** **(1b):** *Step b*. Pyridine (3.76 g, 47.55 mmol) was added to a solution of 2-(1,1-diethoxyethyl)phenanthrene (**1a)** (7.0 g, 23.77 mmol) in chloroform (50 mL) and stirred at 0^o^C for 5 min. Then the dilute solution of trifluoroacetic anhydride (9.98 g, 47.55 mmol) in 20 mL of chloroform was added dropwise over 15 min. The resulting solution was stirred at room temperature for 5 to 6 h; after completion of the reaction monitored by TLC, ice cold water was added and extracted in DCM, and the DCM extract was washed with 2 N HCl solution, then with 10% aq.Na_2_CO_3_ solution, then combined organic extracts were dried over anhydrous Na_2_SO_4_. The solvent was evaporated under reduced pressure to give the crude product. Further purification involved flash column chromatography to give (Z)-4-ethoxy-1,1,1-trifluoro-4-(phenanthren-2-yl)but-3-en-2-one **(1b)** (6.95 g) 85% yield. ^1^H NMR (400 MHz, CDCl_3_) δ: 8.70~ 8.67 (m, 2H), 8.10 (s, 1H), 7.94 ~ 7.89 (m, 1H), 7.82 ~ 7.73 (m, 3H), 7.69 ~ 7.63 (m, 2H), 5.91 (s, 1H), 4.25 (q, *J* = 7.0 Hz, 2H), 1.54 (t, *J* = 7.0 Hz, 3H).

**4-Ethoxy-4-(phenanthren-2-yl)-2-(trifluoromethyl)-2-((trimethylsilyl)oxy)but-3-enenitrile** **(1c):** *Step c.* To a solution of trimethylsilyl cyanide (5.0 g, 14.5 mmol) and triethylamine (0.073 g, 0.726 mmol) in acetonitrile (20 mL) was added an acetonitrile (20 mL) solution of **(**Z)-4-ethoxy-1,1,1-trifluoro-4-(phenanthren-2-yl)but-3-en-2-one **(1b)** at 0 ^o^C, and the resulting solution was stirred for 16 h and concentrated. Further purification involved flash column chromatography to give (Z)-4-ethoxy-4-(phenanthren-2-yl)-2-(trifluoromethyl)-2-((trimethylsilyl)oxy)but-3-enenitrile **(1c)** (4.89 g) 76% yield. ^1^H NMR (400 MHz, CDCl_3_) δ: 8.72 ~ 8.68 (m, 2H), 7.96 ~ 7.88 (m, 2H), 7.78 ~ 7.75 (m, 2H), 7.68 ~ 7.62 (m, 3H), 4.89 (s, 1H), 3.96 (q, *J* = 6.8 Hz, 2H), 1.41 (t, *J* =6.8 Hz, 3H), 0.1 (s, 9H).

**2-(Aminomethyl)-4-ethoxy-1,1,1-trifluoro-4-(phenanthren-2-yl)but-3-en-2-ol** (**1d**): *Step d.* To the suspension of LiAlH_4_ (0.753 g, 19.0 mmol) in dry ether (50 mL), a solution of give (Z)-4-ethoxy-4-(phenanthren-2-yl)-2-(trifluoromethyl)-2-((trimethylsilyl)oxy)but-3-enenitrile **(1c)** (8.0 g, 18.0 mmol) in dry ether (30 mL) was added dropwise with stirring for 30 min at 0–5^o^C. The mixture was stirred overnight at room temperature. Excess LiAlH_4_ was decomposed with 30% aq. NaOH (10 mL) with stirring at 0 ^o^C, then precipitated alumina was filtered and washed thoroughly with ethyl acetate (3 x 50 mL). The filtrate was dried over Na_2_SO_4_ and concentrated under reduced pressure to give an almost pure-white solid amino alcohol (Z)-2-(aminomethyl)-4-ethoxy-1,1,1-trifluoro-4-(phenanthren-2-yl)but-3-en-2-ol (**1d**) (5.28 g, 78% yield). ^1^H NMR (500 MHz, CDCl_3_) δ: 8.68 (d, *J* = 8.4 Hz, 2H), 7.97 (d, *J* = 1.4 Hz, 1H), 7.93 ~ 7.88 (m, 2H), 7.78 ~ 7.60 (m, 5H), 4.88 (s, 1H), 3.93 ~ 3.89 (m, 2H), 2.91 (d, *J* = 13.1 Hz, 1H), 2.61 (d, *J* = 13.6 Hz, 1H), 1.63 (bs, 2H), 1.39 ~ 1.36 (m, 3H). HRMS (ESI): calcd for C_21_H_22_NO_2_F_3_, [M + H] 376.1519, found 376.1524.

**2-(Phenanthren-2-yl)-4-(trifluoromethyl)-1H-pyrrole (1e)**: *Step e*. 5% aq. HCl (1.15 mL, 32.0 mmol) was added to a solution of amino alcohols (Z)-2-(aminomethyl)-4-ethoxy-1,1,1-trifluoro-4-(phenanthren-2-yl)but-3-en-2-ol (**1d**) (3.0 g, 7.99 mmol) in a mixture of acetonitrile (20 mL) and water (1.5 mL). The reaction mixture was stirred at 80^o^C for 12 h. The reaction was monitored by TLC; after completion of the reaction, water and 3% NaHCO_3_ (20 mL) was added, the products were extracted with ethyl acetate (3x 50 mL) and the combined organics were dried over anhydrous Na_2_SO_4._ After the solvent was evaporated, the crude product was purified by column chromatography (hexane / ethyl acetate= 6 / 2) to give 2-(phenanthren-2-yl)-4-(trifluoromethyl)-1H-pyrrole (**1e**) as an off-white solid (2.13 g, 86% yield). ^1^H NMR (500 MHz, CDCl_3_) δ: 8.80 (s, 1H), 8.69 (d, *J* = 8.4 Hz, 1H), 8.65 (d, *J* = 8.2 Hz, 1H), 7.95(s, 1H), 7.89 (d, *J* = 7.7 Hz, 1H), 7.78 ~ 7.76 (m, 2H), 7.73 ~ 7.72 (m, 1H), 7.69 ~ 7.65 (m, 1H), 7.62 ~ 7.59 (m, 1H), 7.22 (s, 1H), 6.83 (s, 1H).

**1-(4-Nitrophenyl)-2-(phenanthren-2-yl)-4-(trifluoromethyl)-1H-pyrrole (1f):** *Step f*. To a solution of 2-(phenanthren-2-yl)-4-(trifluoromethyl)-1H-pyrrole (**1e**) (2.0 g, 6.42 mmol) in DMF (20 mL), 4-Fluoro Nitrobenzene (1.35g, 9.63 mmol) and K_2_CO_3_ (1.77 g, 12.8 mmol) was added. The reaction mixture was stirred at 120^o^C temperature for 12 h. The reaction was monitored by TLC and after completion, ice cold water was added, the product were extracted with ethyl acetate (3x 50 mL) and the combined organic solvent dried was over anhydrous Na_2_SO_4._  After the solvent was evaporated, the crude product was purified by column chromatography (hexane/ethyl acetate= 7/3) to give 1-(4-nitrophenyl)-2-(phenanthren-2-yl)-4-(trifluoromethyl)-1H-pyrrole (**1f**) as a light yellow solid (1.80 g, 65% yield). ^1^H NMR (500 MHz, CDCl_3_) δ: 8.55 (d, *J* = 8.1 Hz, 1H), 8.50 (d, *J* = 8.6 Hz, 1H), 8.11 (d, *J* = 8.8 Hz, 2H), 7.84 (d, *J* = 7.5 Hz, 1H), 7.71 ~ 7.67 (m, 2H), 7.63 ~ 7.55 (m, 3H), 7.30 ~ 7.22 (m, 4H), 6.74 (s, 1H); ^13^C NMR (125 MHz, CDCl_3_) δ: 146.6, 144.5, 135.2, 132.3, 132.1, 129.9, 129.7, 129.0, 128.7, 128.4, 127.9, 127.1, 126.9, 126.7, 126.5, 125.9, 124.8, 123.1, 122.8, 122.7, 117.4 (d, *J* = 37.5 Hz), 109.3.

**4-(2-(Phenanthren-2-yl)-4-(trifluoromethyl)-1H-pyrrol-1-yl)aniline** (**1g**) : *Step g*. 10% palladium on carbon (122 mg, 1.15 mmol) was added to a solution of 1-(4-nitrophenyl)-2-(phenanthren-2-yl)-4-(trifluoromethyl)-1H-pyrrole (**1f**) (1.0 g, 2.31 mmol) in (20 ml) of ethyl acetate and (10 mL) of methanol; the reaction was carried out at 70 PSI (H_2_ gas) on the Par apparatus for 3 h. After completion, the reaction was filtered through celite to remove the catalyst and concentrated to dryness under vacuum to give pure **4**-(2-(phenanthren-2-yl)-4-(trifluoromethyl)-1H-pyrrol-1-yl)aniline (**1g**) (725 mg, 78 % yield). ^1^H NMR (400 MHz, CDCl_3_) δ: 8.59 (d, *J* = 8.4 Hz, 1H), 8.48 (d, *J* = 8.8 Hz, 1H), 7.86 (d, *J* = 7.6 Hz, 1H), 7.72 ~ 7.69 (m, 2 H), 7.64 ~ 7.55 (m, 2H), 7.35 (dd, *J* = 8.8, 1.6 Hz, 1H), 7.22 (s, 1H), 6.98 (d, *J* = 8.4 Hz, 2H), 6.80 (d, *J* = 8.8 Hz, 2H), 6.62 ~ 6.61(m, 2H), 3.75 (s, 2H).

**Method** : **Synthesis of representative Pyrrole derivatives with different Amino Acids using PyBOP:** PyBOP (3.2 eq.) was added to an ice-cold solution of individual amino acid (3.0 equiv) and TEA (3.0 equiv) in anhydrous DMF. After being stirred for 1 h at 25^o^C, the reaction mixture was cooled to 0 ºC and amine **(1g)** (1.0 eq.) and triethylamine (3.0 equiv) was added. The resulting mixture was stirred at for 48 h at 25 ^o^C, and reaction progress was monitored by TLC. After completion, the reaction mixture was poured into ice-cold water, extracted with ethyl acetate (2 X 50 mL), a combined organic solvent was dried over sodium sulfate, filtered and concentrated. The residue was purified by flash column chromatography (CH_2_Cl_2_ / MeOH) to give corresponding Boc protected amide in 70-79% yield. This Boc protected amide (0.200 g) was dissolved in 10 ml of ethyl acetate containing 2.0 ml of concentrated HCl solution, stirred at room temperature for 2 h, and concentrated to dryness under reduced pressure. The crude product was purified by silica gel column chromatography (DCM / Methanol / NH_4_OH = 96 / 2 / 2) to give corresponding derivatives in 54-83% yield.

**2-Amino-2-methyl-*N*-(4-(2-(phenanthren-2-yl)-4-(trifluoromethyl) -1*H*-pyrrol-1-yl) phenyl) propanamide (P12-23):** ^1^H NMR (400 MHz, CDCl_3_) δ: 9.95 (s, 1H), 8.48 (d, *J* = 8.0 Hz, 1H), 8.39 (d, *J* = 8.8 Hz, 1H), 7.78 (d, *J* = 7.2 Hz, 1H), 7.67 ~ 7.62 (m, 2H), 7.54 ~ 7.50 (m, 5H), 7.23 ~ 7.19 (m, 3H), 7.09 ~ 7.07 (m, 2H), 6.69 (s, 1H), 1.36 (s, 6H); ^13^C NMR (100 MHz, CDCl_3_) δ:175.9, 137.6, 134.8, 134.7, 131.9, 131.8, 129.8, 129.7, 129.1, 128.5, 127.9, 127.4, 126.8, 126.7, 126.3, 123.2, 122.6, 119.7, 116.0, 115.5, (d, *J* = 37.0 Hz), 107.6, 55.2, 28.9; HRMS (ESI): calcd for C_29_H_25_N_3_OF_3_, [M + H] 488.1950, found 488.1953.

**(*S*)-2-amino-*N*-(4-(2-(phenanthren-2-yl)-4-(trifluoromethyl)-1*H*-pyrrol-1-yl)phenyl)-3-phenylpropanamide (P12-34):** ^1^H NMR (400 MHz, CDCl_3_) δ: 9.52 (s, 1H), 8.55 (d, *J* = 8.0 Hz, 1H), 8.44 (d, *J* = 8.8 Hz, 1H), 7.82 (d, *J* = 7.6 Hz, 1H), 7.69 ~ 7.67 (m, 2H), 7.59 ~ 7.54 (m, 5H), 7.31 ~ 7.12 (m, 10H), 6.71 (s, 1H), 3.67 (d, *J* = 6.8 Hz, 1H), 3.32 (d, *J* = 11.6 Hz, 1H), 2.76 ~ 2.70 (m, 1H); ^13^C NMR (100 MHz, CDCl_3_) δ: 172.5, 137.4, 137.2, 135.1, 134.7, 131.9, 131.8, 129.9, 129.7, 129.2, 129.1, 128.8, 128.5, 127.9, 127.4, 127.0, 126.9, 126.8, 126.7,126.4, 123.2, 122.6 (2xCH), 119.9, 115.7 (d, *J* = 37.0 Hz), 107.6, 56.6, 40.5. HRMS (ESI): calcd for C_34_H_27_N_3_OF_3_, [M + H] 550.2106, found 550.2101; Specific optical rotation [α]^20^_D_ - 17.0° (CHCl_3_).

**General synthetic procedure for Synthesis of biotinylated P12-34:**

**Synthesis of 2-chlorophenanthren-9-ol (2):**  2-chlorophenanthren-9-ol (**2**) was prepared from commercially available 2-(2-bromo-5-chlorophenyl)acetic acid (**1**) over five synthetic steps following a similar procedure reported previously (PCT Int. Appl. 2007095753, 30 Aug 2007).

**Synthesis of 2-chloro-9-methoxyphenanthrene (3):** Potassium hydroxide (1.10 g, 19.68 mmol) was added to a solution of 2-chlorophenanthren-9-ol (**2**) (3.00 g, 13.12 mmol) in acetone (30 mL), followed by dimethyl sulfate (1.86 mL, 19.68 mmol). The resulting mixture was refluxed for 2 h under argon. After completion of reaction (TLC), volatiles were removed under reduced pressure; the obtained residue was dissolved in between water (100 mL) and ethyl acetate (100 mL). The organic layer was separated, washed with water (50 mL), brine (50 mL), and dried over anhydrous sodium sulfate, then concentrated under reduced pressure*.* The obtained residue was purified by flash column chromatography (silica gel, hexanes: ethyl acetate) to provide 2-chloro-9-methoxyphenanthrene (**3**) (2.87 g, 11.86 mmol, 90%). ^1^H NMR (300 MHz, CDCl_3_) δ: 8.56 (dd, *J* = 8.4, 1.0 Hz, 1H), 8.47 (d, *J* = 8.9 Hz, 1H), 8.35 (dd, *J* = 7.6, 1.7 Hz, 1H), 7.73 (d, *J* = 2.2 Hz, 1H), 7.72 ~ 7.58 (m, 2H), 7.43 (dd, *J* = 8.8, 2.2 Hz,1H), 6.87 (s, 1H), 4.08 (s, 1H).

**Synthesis of triisopropyl((9-methoxyphenanthren-2-yl)ethynyl) silane (4):** To a degassed solution of 2-chloro-9-methoxyphenanthrene (**3**) (2.80 g, 11.54 mmol) in acetonitrile (40 mL) was added (triisopropylsilyl) acetylene (2.75 g, 15.00 mmol), cesium carbonate (7.50 g, 23.08 mmol), xphos (0.275 g, 0.58 mmol) and bis(triphenylphosphine) palladium(II) dichloride (0.217 g, 0.29 mmol) and the resulting mixture was refluxed for 14 h. After completion of the reaction (TLC), reaction mixture was cooled, diluted with ethyl acetate (100 mL) and washed with water (2 × 50 mL), brine (50 mL). The organic layer was dried over anhydrous sodium sulfate and concentrated under reduced pressure*.* The residue was purified by flash column chromatography (silica gel, hexanes: ethyl acetate) to provide triisopropyl ((9-methoxyphenanthren-2-yl) ethynyl) silane (**4**) (2.87 g, 7.38 mmol, 62%). ^1^H NMR (300 MHz, CDCl_3_) δ: 8.60 (d, *J* = 7.6 Hz, 1H), 8.48 (d, *J* = 8.6 Hz, 1H), 8.35 (d, *J* = 8.7 Hz, 1H), 7.92 (d, *J* = 1.5 Hz, 1H), 7.70 ~ 7.60 (m, 2H), 7.56 (dd, *J* = 8.4, 1.7 Hz, 1H), 6.93 (s, , 1H), 4.08 (s, 3H), 1.18 (s, 21H).

**Synthesis of 2-ethynyl-9-methoxy phenanthrene (5):** A solution of TBAF in THF (8.12 mL, 8.12 mmol) was added to a cooled (0 °C) solution of triisopropyl ((9-methoxyphenanthren-2-yl) ethynyl) silane (**4**) (2.87 g, 7.38 mmol) in THF (30 mL). The resulting mixture was stirred at the same temperature for 30 min. After completion of the reaction (TLC), the reaction mixture was cooled, diluted with ethyl acetate (100 mL) and washed with water (2 × 50 mL) and brine (50 mL). The organic layer was dried over anhydrous sodium sulfate, then concentrated under reduced pressure to give 2-ethynyl-9-methoxyphenanthrene (**5**) (1.70 g, 7.32 mmol, 99%). ^1^H NMR (300 MHz, CDCl_3_) δ: 8.60 (dd, *J* = 8.4, 0.6 Hz, 1H), 8.53 (d, *J* = 8.5 Hz, 1H), 8.36 (dd, *J* = 8.0, 1.3 Hz, 1H), 7.95 (d, *J* = 1.4Hz, 1H), 7.72 ~ 7.62 (m, 2H), 7.57 (dd, *J* = 8.5, 1.7 Hz, 1H), 6.93 (s, 1H), 4.09 (s, 3H), 3.17 (s, 1H); HRMS (ESI): calcd for C_17_H_13_O^+^, [M + H^+^] 233.0966, found 233.0965.

**Synthesis of 9-methoxy-2-(3-(trifluoromethyl) but-3-en-1-yn-1-yl) phenanthrene (6):** To a degassed solution of 2-ethynyl-9-methoxy phenanthrene (**5**) (6.00 g, 25.83 mmol) in anhydrous THF was added 2-bromo-3,3,3-trifluoro-1-propene (5.42 g, 31.00 mmol), triethyamine (11 mL, 77.48 mmol), CuI (0.49 g, 2.58 mmol) and tetrakis (triphenylphosphine) palladium (0) (1.20 g, 1.03 mmol) in a sealed tube. The resulting mixture was stirred at 60 °C for 14 h. After completion of the reaction (TLC), the reaction mixture was cooled, diluted with ethyl acetate (100 mL) and filtered through a pad of celite. The filtrate was concentrated under reduced pressure. The obtained residue was purified by flash column chromatography (silica gel, hexanes: ethyl acetate) to provide 9-methoxy-2-(3-(trifluoromethyl) but-3-en-1-yn-1-yl) phenanthrene (**6**) (6.30 g, 19.31 mmol, 75%). ^1^H NMR (400 MHz, CDCl_3_) δ: 8.61 (d, *J* = 7.9, 0.4 Hz, 1H), 8.53 (d, *J* = 8.5 Hz, 1H), 8.36 (dd, *J* = 8.0, 1.3 Hz, 1H), 7.95 (d, *J* = 1.4 Hz, 1H), 7.72 ~ 7.62 (m, 2H), 7.57 (dd, *J* = 8.5, 1.7 Hz, 1H), 6.93 (s, 1H), 6.15 (d, *J* = 1.2 Hz, 1H), 6.00 (d, *J* = 1.3 Hz, 1H), 4.09 (s, 3H). HRMS (ESI): calcd for C_20_H_14_F_3_O^+^, [M + H^+^] 327.0991, found 327.0996.

**Synthesis of *N*-(4-(9-methoxyphenanthren-2-yl) -2-(trifluoromethyl) but-3-yn-1-yl) hydroxyl amine (7):** Triethylamine (8.1 mL) and hydroxylamine hydrochloride (2.68 g, 38.62 mmol) was added portion wise to a cooled (0 °C) solution of 9-methoxy-2-(3-(trifluoromethyl) but-3-en-1-yn-1-yl) phenanthrene (**6**) (6.30 g, 19.31 mmol) in anhydrous dichloromethane (60 mL), the reaction mixture was stirred at 0 °C to room temperature for 14 h. After completion of the reaction (TLC), the reaction mixture was cooled, diluted with the dichloromethane (100 mL), washed with water (50 mL) and brine (50 mL), dried over anhydrous sodium sulfate and concentrated under reduced pressure. The obtained residue was purified by column chromatography (silica gel, hexanes : ethyl acetate) to give *N*-(4-(9-methoxy phenanthren-2-yl)-2-(trifluoromethyl) but-3-yn-1-yl) hydroxylamine (**7**) (4.72 g, 13.13 mmol, 68%). ^1^H NMR (400 MHz, CDCl_3_) δ: 8.60 (d, *J* = 8.2 Hz, 1H), 8.50 (d, *J* = 8.8 Hz, 1H), 8.35 (dd, *J* = 8.4, 1.6 Hz, 1H), 7.92 (d, *J* = 1.6 Hz, 1H), 7.71 ~ 7.62 (m, 2H), 7.54 (dd, *J* = 8.5, 1.7 Hz, 1H), 6.92 (s, 1H), 5.66 (br s, 1H), 4.88 (br s, 1H), 4.12 ~ 3.98 (m, 4H), 3.50 (dd, *J* = 13.2, 4.4 Hz, 1H), 3.23 (dd, *J* = 13.2, 9.4 Hz, 1H); HRMS (ESI): calcd for C_20_H_17_F_3_NO_2_^+^, [M + H^+^] 360.12059, found 360.12057.

**Synthesis of 2-(9-methoxy phenanthren-2-yl)-4-(trifluoromethyl)-1*H*-pyrrole (8):** AgNTf_2_ (66 mg, 0.17 mmol) was added to a cooled (0 °C) solution of IPrAuCl (108 mg, 0.17 mmol) in anhydrous DMF (1.5 mL) and the mixture was stirred at same temperature for 10 min. A solution of *N*-(4-(9-methoxy phenanthren-2-yl)-2-(trifluoromethyl) but-3-yn-1-yl) hydroxyl amine (**7**) (1.25 g, 3.48 mmol) in anhydrous DMF (2 mL) and HNTf_2_ (98 mg, 0.35 mmol) was added at room temperature. The resulting mixture was stirred at room temperature for 14 h. After completion of the reaction (TLC), the reaction mixture was diluted with water (30 mL) and extracted with ethyl acetate (3 × 30 mL). The combined organic layers were washed with brine (50 mL), dried over anhydrous sodium sulfate and concentrated under reduced pressure. The obtained residue was purified by flash column chromatography (silica gel, hexanes: ethyl acetate) to give 2-(9-methoxy phenanthren-2-yl)-4-(trifluoro methyl)-1*H*-pyrrole (**8**) (0.62 g, 1.81 mmol, 52%). ^1^H NMR (400 MHz, CDCl_3_) δ: 8.73 (br s, 1H), 8.61 (d, *J* = 7.8 Hz, 1H), 8.57 (d, *J* = 8.7 Hz, 1H), 8.36 (dd, *J* = 8.2, 1.2 Hz, 1H), 7.84 (d, *J* = 2.0 Hz, 1H), 7.71 ~ 7.67 (m, 1H), 7.64 ~ 7.58 (m, 2H), 7.22 ~ 7.20 (m, 1H), 6.97 (s, 1H), 6.81(s, 1H), 4.10 (s, 1H).

**Synthesis of 2-(9-methoxy phenanthren-2-yl)-1-(4-nitrophenyl)-4-(trifluoromethyl)-1*H*-pyrrole (9):** Potassium carbonate (1.82 g, 12.90 mmol) and 1-fluoro-4-nitrobenzene (1.78 g, 12.90 mmol) were added to a solution of 2-(9-methoxy phenanthren-2-yl)-4-(trifluoromethyl)-1*H*-pyrrole (**8**) (2.20 g, 6.45 mmol) in anhydrous DMF (15 mL). The resulting mixture was stirred at 130 ^o^C for 14 h. After completion of the reaction (TLC), the reaction mixture was diluted with ethyl acetate (100 mL) and water (50 mL). The organic layer was separated, washed with water (3 × 50 mL) and brine (2 × 50 mL), dried over anhydrous sodium sulfate and concentrated under reduced pressure. The obtained residue was purified by column chromatography (silica gel, hexanes : ethyl acetate) to give 2-(9-methoxy phenanthren-2-yl)-1-(4-nitrophenyl)-4-(trifluoromethyl)-1*H*-pyrrole (**9**) (1.73 g, 3.74 mmol, 58%). ^1^H NMR (300 MHz, CDCl_3_) δ: 8.55 (d, *J* = 8.6 Hz, 1H), 8.42 (d, *J* = 8.7 Hz, 1H), 8.35 (dd, *J* = 7.6, 1.8 Hz, 1H), 8.20 (dt, *J* = 9.6, 2.5 Hz, 2H), 7.70 ~ 7.60 (m, 3H), 7.39 ~ 7.34 (m, 3H), 7.09 (dd, *J* = 8.5, 1.9 Hz, 1H), 6.85(s, 1H), 6.75 (d, *J* = 1.40 Hz, 1H), 4.07(s, 3H); HRMS (ESI): calcd for C_26_H_18_F_3_N_2_O_3_^+^, [M + H^+^] 463.12640, found 463.12643.

**Synthesis of 2-(1-(4-nitrophenyl)-4-(trifluoromethyl)-1*H*-pyrrol-2-yl) phenanthren-9-ol (10):** 33% HBr in acetic acid (12 mL) was added to a 2-(9-methoxy phenanthren-2-yl)-1-(4-nitrophenyl)-4-(trifluoromethyl)-1*H*-pyrrole (**9**) (1.73 g, 3.74 mmol) and the resulting solution was stirred at 90 °C for 14 h. After completion of the reaction (TLC), the reaction mixture was concentrated under reduced pressure; the obtained residue was treated with saturated aqueous solution of sodium carbonate to adjust pH to ~ 8. The aqueous layer was extracted with ethyl acetate (3 × 30 mL). The combined organic layers were washed with brine (2 × 30 mL), dried over anhydrous sodium sulfate and concentrated under reduced pressure. The obtained residue was purified by flash column chromatography (silica gel, hexanes: ethyl acetate) to give 2-(1-(4-nitrophenyl)-4-(trifluoromethyl)-1*H*-pyrrol-2-yl)phenanthren-9-ol (**10**) (1.21 g, 2.70 mmol, 72%). ^1^H NMR (300 MHz, CDCl_3_) δ: 8.58 (dd, *J* = 7.5, 1.9 Hz, 1H), 8.44 (d, *J* = 8.7 Hz, 1H), 8.31 (dd, *J* = 7.2, 2.3 Hz, 1H), 7.21 ~ 7.16 (m, 2H), 7.72 ~ 7.63 (m, 2H), 7.52 (d, *J* = 1.9 Hz, 1H), 7.37 ~ 7.32 (m, 3H), 7.12 (dd, *J* = 8.6, 1.9 Hz, 1H), 6.88 (s, 1H), 6.75 (d, *J* = 1.4 Hz, 1H), 5.72 (s, 1H).

**Synthesis of benzyl (2-((2-(1-(4-nitrophenyl) -4-(trifluoromethyl) -1*H*-pyrrol-2-yl) phenanthren-9-yl)oxy) ethyl) carbamate (11):** To a cooled (0 °C) slurry of NaH (98 mg, 2.45 mmol) in anhydrous DMF (3 mL) was added solution of 2-(1-(4-nitrophenyl) -4-(trifluoromethyl) -1*H*-pyrrol-2-yl) phenanthren-9-ol (**10**) (0.50 g, 1.12 mmol) in anhydrous DMF (4 mL) slowly and the resulting mixture was stirred at room temperature for 30 min. A solution of benzyl (2-bromoethyl)carbamate (0.43 g, 1.67 mmol) in anhydrous DMF ( 3 mL) was added slowly. The reaction mixture was stirred at 60 °C for 14 h. After completion of the reaction (TLC), the reaction mixture was cooled to room temperature and quenched with the addition of cold water (20 mL). The reaction mixture was extracted with ethyl acetate (3 × 35 mL). The combined organic layers were washed with water (2 × 30 mL) and brine (2 × 30 mL), dried over anhydrous sodium sulfate, and concentrated under reduced pressure. The obtained residue was purified by flash column chromatography (silica gel, hexanes: ethyl acetate) to give benzyl (2-((2-(1-(4-nitrophenyl)-4-(trifluoromethyl)-1*H*-pyrrol-2-yl)phenanthren-9-yl)oxy)ethyl)carbamate (**11**) (0.35 g, 0.56 mmol, 50%). ^1^H NMR (300 MHz, CDCl_3_) δ: 8.56 (d, *J* = 8.6, 1H), 8.43 (d, *J* = 8.7 Hz, 1H), 8.31 (dd, *J* = 8.0, 1.4 Hz, 1H), 8.22 ~ 8.17 (m, 2H), 7.71 ~ 7.60 (m, 3H), 7.43 ~ 7.28 (m, 9H), 7.71 ~ 7.60 (m, 3H), 7.41 ~ 7.28 (m, 9H), 7.12 (dd, *J* = 8.5, 1.9 Hz, 1H), 6.84 (s, 1H), 6.75 (d, *J* = 1.3Hz, 1H), 5.27 (br s, 1H), 5.14 (s, 2H), 4.30 (t, *J* = 5.0 Hz, 2H), 3.81 (q, *J* = 5.3 Hz, 2H).

**Synthesis of benzyl (2-((2-(1-(4-aminophenyl) -4-(trifluoromethyl) -1*H*-pyrrol-2-yl) phenanthren-9-yl)oxy) ethyl) carbamate (12):** Fe powder (0.31 g, 5.60 mmol) was added to a solution of benzyl (2-((2-(1-(4-nitrophenyl) -4-(trifluoromethyl) -1*H*-pyrrol-2-yl) phenanthren-9-yl) oxy) ethyl) carbamate (**11**) (0.35 g, 0.56 mmol) in ethanol (7 mL) and water (7 mL), followed by ammonium chloride (0.30 g, 5.60 mmol) and the resulting mixture was refluxed for 2 h. After completion of reaction (TLC), reaction mixture was cooled and diluted with ethyl acetate (70 mL) and water (30 mL). The organic layer was separated, aqueous layer was extracted with ethyl acetate (2 × 30 mL). The combined organic layers were washed with brine (30 mL), dried over anhydrous sodium sulfate and concentrated under reduced pressure to obtain benzyl (2-((2-(1-(4-aminophenyl)-4-(trifluoromethyl)-1*H*-pyrrol-2-yl)phenanthren-9-yl)oxy)ethyl)carbamate (**12**) and used as is without further purification in next step (0.33 g, 5.60 mmol, quant.).

**Synthesis of benzyl (*S*)-(2- ((2-(1-(4-(2-( (*tert*-butoxycarbonyl) amino) -3-phenyl propanamido) phenyl) -4-(trifluoro methyl) -1*H*-pyrrol-2-yl) phenanthren-9-yl)oxy) ethyl) carbamate (13):** HATU (0.38 g, 1.01 mmol) and Hunig’s base (0.26 mL, 1.51 mmol) were added at room temperature to a solution of (*tert*-butoxycarbonyl) -*L*-phenylalanine (0.27 g, 1.01 mmol) in anhydrous DMF (2 mL) and the resulting mixture was stirred for 10 min. A solution of benzyl (2-((2-(1- (4-aminophenyl) -4-(trifluor omethyl) -1*H*-pyrrol-2-yl) phenanthren-9-yl)oxy) ethyl) carbamate (**12**) (0.30 g, 50 mmol) in anhydrous DMF (2 mL) was added slowly and the reaction mixture was stirred at room temperature for 14 h. After completion of the reaction (TLC), the reaction mixture was diluted with ethyl acetate (50 mL) and water (20 mL). The organic layer was separated, and the aqueous layer was extracted with ethyl acetate (2 × 20 mL). The combined organic layers were washed with brine (30 mL), dried over anhydrous sodium sulfate and concentrated under reduced pressure. The obtained residue was purified by flash column chromatography (silica gel, hexanes: ethyl acetate) to give benzyl (*S*)-(2-((2- (1-(4- (2-((*tert*-butoxycarbonyl) amino) -3-phenyl propanamido) phenyl)-4-(trifluoromethyl) -1*H*-pyrrol-2-yl) phenanthren-9-yl)oxy) ethyl) carbamate (**13**) (0.25 g, 0.30 mmol, 30%). ^1^H NMR (400 MHz, CDCl_3_) δ: 8.54 (d, *J* = 8.2, 1H), 8.38 (d, *J* = 8.7 Hz, 1H), 8.29 (d, *J* = 8.2 Hz, 1H), 7.94 ~ 7.84 (m, 1H), 7.69 ~ 7.56 (m, 2H), 7.54 (s, 1H), 7.39 ~ 7.27 (m, 9H), 7.25 ~ 7.19 (m, 4H), 7.19 ~ 7.10 (m, 3H), 6.80 (s, 1H), 6.70 (d, *J* = 1.5 Hz, 1H), 5.30 (brs, 1H), 5.23 (brs, 3H), 4.43 (s, 1H), 4.30 (t, *J* = 9.5 Hz, 2H), 3.80 (t, *J* = 5.2 Hz, 2H), 3.14 (t, *J* = 7.0 Hz, 2H), 1.39 (s, 9H).

**Synthesis of *tert*-butyl (*S*)-(1- ((4-(2- (9-(2-amino ethoxy) phenanthren-2-yl)-4-(trifluoro methyl) -1*H*-pyrrol-1-yl) phenyl)amino) -1-oxo-3-phenylpropan-2-yl) carbamate (14):** Pd(OH)_2_ on carbon (30 mg) was added to a solution of benzyl (*S*)-(2-((2-(1-(4-(2-((*tert*-butoxycarbonyl) amino)-3-phenyl propanamido) phenyl)-4-(trifluoro methyl)-1*H*-pyrrol-2-yl) phenanthren-9-yl)oxy) ethyl) carbamate (**13**) (0.25 g, 0.30 mmol) in ethyl acetate (3 mL) and methanol (3 mL); the resulting mixture was stirred under H_2_ (50 psi) atmosphere for 2 h in a Parr hydrogenator. The reaction mixture was filtered through bed of celite, washed with ethyl acetate; the filtrate was concentrated to obtain *tert*-butyl (*S*)-(1-((4-(2-(9- (2-amino ethoxy) phenanthren-2-yl) -4-(trifluoromethyl)-1*H*-pyrrol-1-yl) phenyl) amino)-1-oxo-3-phenylpropan-2-yl) carbamate (**14**) and used as is without further purification in the next step (0.21 g, 0.30 mmol, quant.); HRMS (ESI): calcd for C_41_H_40_F_3_N_4_O_4_^+^, [M + H^+^] 709.29962, found 709.30048.

**Synthesis of benzyl ((2*S*)-1-((4-(2-(9- ((4,44-dioxo-48- (2-oxohexa hydro-1*H*-thieno [3,4-d] imidazol- 4-yl)-7,10,13,16,19,22,25,28,31,34,37,40 -dodecaoxa -3,43-diaza octa tetracontyl) oxy) phenanthren -2-yl)-4-(trifluoro methyl)-1*H*-pyrrol-1-yl) phenyl) amino) -1-oxo -3-phenyl propan -2-yl) carbamate (15):** Triethylamine (12 µl, 0.08 mmol) was added to a solution of *tert*-butyl (*S*)-(1-((4-(2-(9- (2-amino ethoxy) phenanthren-2-yl) -4-(trifluoro methyl)-1*H*-pyrrol-1-yl) phenyl) amino)-1-oxo-3-phenyl propan-2-yl) carbamate (**14**) (30 mg, 0.04 mmol) in anhydrous DMF (1 mL), followed by a solution of NHS-dPEG 12-biotin (39 mg, 0.04 mmol) in anhydrous DMF (1 mL) slowly and the resulting mixture was stirred at room temperature for 16 h. After completion of the reaction (TLC), the reaction mixture was diluted with ethyl acetate (20 mL) and washed with water (2 × 20 mL), brine (20 mL) and concentered under reduced pressure. The obtained residue was purified by flash column chromatography (silica gel, dichloromethane: methanol) to obtain benzyl ((2*S*)-1-((4-(2-(9-( (4,44-dioxo- 48-(2-oxo hexa hydro-1*H*-thieno[3,4-d] imidazol-4-yl)-7,10,13,16,19,22,25,28,31,34,37,40-dodeca oxa-3,43-diaza octatetracontyl) oxy) phenanthren-2-yl) -4-(trifluoro methyl) -1*H*-pyrrol-1-yl) phenyl) amino)-1-oxo-3-phenyl propan-2-yl) carbamate (**15**) (30 mg, 0.019 mmol, 48%) ^1^H NMR (400 MHz, CDCl_3_) δ: 8.80 (brs, 1H), 8.54 (d, *J* = 8.0 Hz, 1H), 8.38 (d, *J* = 8.8 Hz, 1H), 8.35 (d, *J* = 8.0 Hz, 1H), 7.67 ~ 7.58 (m, 2H), 7.55 (s, 1H), 7.48 (d, *J* = 8.1, 1H), 7.26 ~ 7.06 (m, 8H), 6.83 (s, 1H), 6.69 (s, 1H), 6.60 (br s, 1H), 5.68 (s, 1H), 5.36 (s, 1H), 5.21 (s, 1H), 4.52 (s, 1H), 4.44 (t, 6.1 Hz, 1H) , 4.36 ~ 4.27 (m, 1Hz), 4.27 ~ 4.21 (m, 1H), 3.88 ~ 3.80 (m, 1H), 3.72 (t, *J* = 5.6 Hz, 2H), 3.68 ~ 3.34 (m, 44H), 3.22 ~ 2.97 (m, 4H), 2.86 (m, 1H), 2.68 (d, *J* = 12.7 Hz, 1H), 2.52 (t, *J* = 5.4 Hz, 2H), 2.23 ~ 2.12 (m, 1H), 1.73 ~ 1.50 (m, 6H), 1.36 (s, 9H), 1.24 ~ 1.14 (m, 2H); HRMS (ESI): calcd for C_78_H_106_F_3_N_7_O_19_SNa^+^, [M + Na^+^] 1556.71085, found 1556.70923.

**Synthesis of *N*-(2-((2-(1-(4-((*S*)-2- amino-3-phenyl propanamido) phenyl)-4-(trifluoro methyl) -1*H*-pyrrol-2-yl) phenanthren-9-yl) oxy) ethyl) -1-(5-(2-oxohexa hydro-1*H*-thieno [3,4-d] imidazol-4-yl) pentanamido) -3,6,9,12,15,18,21,24,27,30,33,36- dodecaoxa nona triacontan-39-amide (BP12-34):** A 50% solution of TFA (200 µl) in dichloromethane was added to a cooled (0 °C) solution of benzyl ((2*S*)-1- ((4-(2-(9- ((4,44-dioxo-48-(2-oxohexahydro-1*H*-thieno[3,4-d] imidazol-4-yl)-7,10,13,16,19,22,25,28,31,34,37,40-dodecaoxa-3,43-diaza octatetracontyl) oxy) phenanthren-2-yl)-4-(trifluoromethyl)-1*H*-pyrrol-1-yl)phenyl) amino)-1-oxo-3-phenylpropan-2-yl) carbamate (**15**) (30 mg, 0.019 mmol) in dichloromethane (2 mL) and the resulting mixture was stirred at room temperature for 3 days. After completion of the reaction (LCMS analysis), the reaction mixture was diluted with dichloromethane (25 mL) and washed with water (10 mL), saturated aqueous sodium bicarbonate (10 mL) and brine (10 mL). The organic layer was dried over anhydrous sodium sulfate, then concentrated under reduced pressure. The obtained residue was triturated with ethyl acetate, diethyl ether and hexane to obtain *N*-(2-((2-(1-(4-((*S*)-2-amino-3-phenylpropanamido) phenyl)-4-(trifluoromethyl)-1*H*-pyrrol-2-yl) phenanthren-9-yl) oxy)ethyl) -1-(5-(2-oxohexa hydro-1*H*-thieno [3,4-d] imidazol-4-yl) pentanamido)-3,6,9,12,15,18,21,24,27,30,33,36- dodecaoxa nonatriacontan-39-amide (**16**) (8 mg, 0.006 mmol, 29%). ^1^H NMR (400 MHz, CDCl_3_) δ: 9.65 (br s, 1H), 8.55 (d, *J* = 7.9 Hz, 1H), 8.39 (d, *J* = 8.8 Hz, 1H), 8.35 (d, *J* = 9.3 Hz, 1H), 7.68 ~ 7.58 (m, 4H), 7.41 ~ 7.26 (m, 7H), 7.23 ~ 7.11 (m, 3H), 6.84 (s, 1H), 6.70 (d, *J* = 1.6 Hz, 1H), 6.43 (br, s, 1H), 5.24 (s, 1H), 4.66 (s, 1H), 4.46 (t, *J* = 5.9 Hz, 1H), 4.33 ~ 4.26 (m, 2Hz), 3.90 ~ 3.80 (m, 2H), 3.72 (t, *J* = 5.7 Hz, 2H), 3.69 ~ 3.34 (m, 44H), 3.16 ~ 3.11 (m, 1H), 2.92 ~ 2.87 (m, 1H), 2.83 ~ 2.77 (m, 1H), 2.70 (d, *J* = 12.8 Hz, 1H), 2.52 (t, *J* = 5.7 Hz, 2H), 2.21 ~ 2.18 (m, 2H), 1.75 ~ 1.50 (m, 4H), 1.49 ~ 1.39 (m, 2H); HRMS (ESI): calcd for C_73_H_99_F_3_N_7_O_17_S^+^, [M + H^+^] 1434.67648, found 1434.67639.

**Supplementary figure legends**

**Supplementary figure 1. Antiviral activities of AR-12 and its derivatives in multiple cell lines.** BHK-21 (a), CHME3 (b), Vero (c), and DC-SIGN-expressing THP-1 (d) cells were treated with AR-12, P12-23 or P12-34, and infected with DENV-2-eGFP (MOI 5) for 24 h. eGFP (a-b) and NS3 (c) protein expression was measured by using high-content image analysis system and normalized with vehicle control to determine the relative level of viral infection. Representative data from repeated experiments are shown as mean and SD (n =3). (d) DENV-infection rates of DC-SIGN-expressing THP-1 cells were analyzed by using flow cytometry.

**Supplementary figure 2. P12-23 does not significantly induced ER stress and autophagy.** A549 cells with or without DENV-2 (MOI 5) infection were treated with P12-23 (1 μM), tunicamycin (TM) (5 μg/ml), thapsigargin (TG) (0.5 μM), or chemical chaperon 4-Phenylbutyric acid (4-PBA) (10 mM) for 24 h. Cell lysates were harvested for western blot analysis with antibodies against the indicated proteins.

**Supplementary figure 3. Intracellular distribution of P12-34.** A549 cells were treated with BP12-34, then stained with organelle-specific antibodies [autophagosome: anti-LC3B pAb; ER: anti-calnexin mAb; Golgi: anti-GM130 mAb]. Nuclei were stained with DAPI. Images were acquired by Zeiss LSM700 confocal microscopy. Scale bar = 20 µM.

**Supplementary figure 4. P12-34 interacts with cytochrome bc1 complex.** Cell lysates of A549 were treated with 0.25 μM (+) or 0.5 μM (++) BP12-34 at 4 ℃ for 1 h, then incubated with streptavidin (SA) beads at 4 ℃ for 30 min. Non-specific binding was removed by washing with PBS. After precipitation, P12-34-associated proteins were analyzed by western blot with the indicated antibodies against DHODH and cytochrome bc1 complex.

**Supplementary figure 5. P12-34 amplifies innate immune response.** (a) HEK 293T cells were co-transfected with pISRE-luc and pRL-TK *Renilla* reporter plasmids, and short synthetic 5′-triphosphate RNA molecules (ssRNA), then treated with DMSO, brequinar (1 μM), or P12-34 (1 μM) for 24 h. ISRE-Luciferase activity was normalized with *Renilla*-luciferase activity in each sample, and then the relative ISRE-Luc level was determined with the DMSO control without ssRNA. Representative data from repeated experiments are shown as mean and SD (n =3). (b) HEK 293T cells were transfected with 30 ng/well ssRNA and treated with DMSO, 1 μM brequinar, or 1 μM P12-34 for 24 h. Cell lysates were harvested for western blot analysis with antibodies against IFIT1, IRF-1 and actin.

**References**

1. Tassaneetrithep, B. *et al.* DC-SIGN (CD209) mediates dengue virus infection of human dendritic cells. *J Exp Med* **197**, 823-829 (2003).

2. Liang, J.J., Liao, C.L., Liao, J.T., Lee, Y.L. & Lin, Y.L. A Japanese encephalitis virus vaccine candidate strain is attenuated by decreasing its interferon antagonistic ability. *Vaccine* **27**, 2746-2754 (2009).

3. Schoggins, J.W. *et al.* Dengue reporter viruses reveal viral dynamics in interferon receptor-deficient mice and sensitivity to interferon effectors in vitro. *Proc Natl Acad Sci U S A* **109**, 14610-14615 (2012).
