# Supplementary figures and images for: Novel AR-12 derivatives, P12-23 and P12-34, inhibit flavivirus replication by blocking host de novo pyrimidine biosynthesis
Source: Emerg Microbes Infect. 2018 Nov 21;7:187. doi: 10.1038/s41426-018-0191-1 (PMC6246607; doi:10.1038/s41426-018-0191-1)

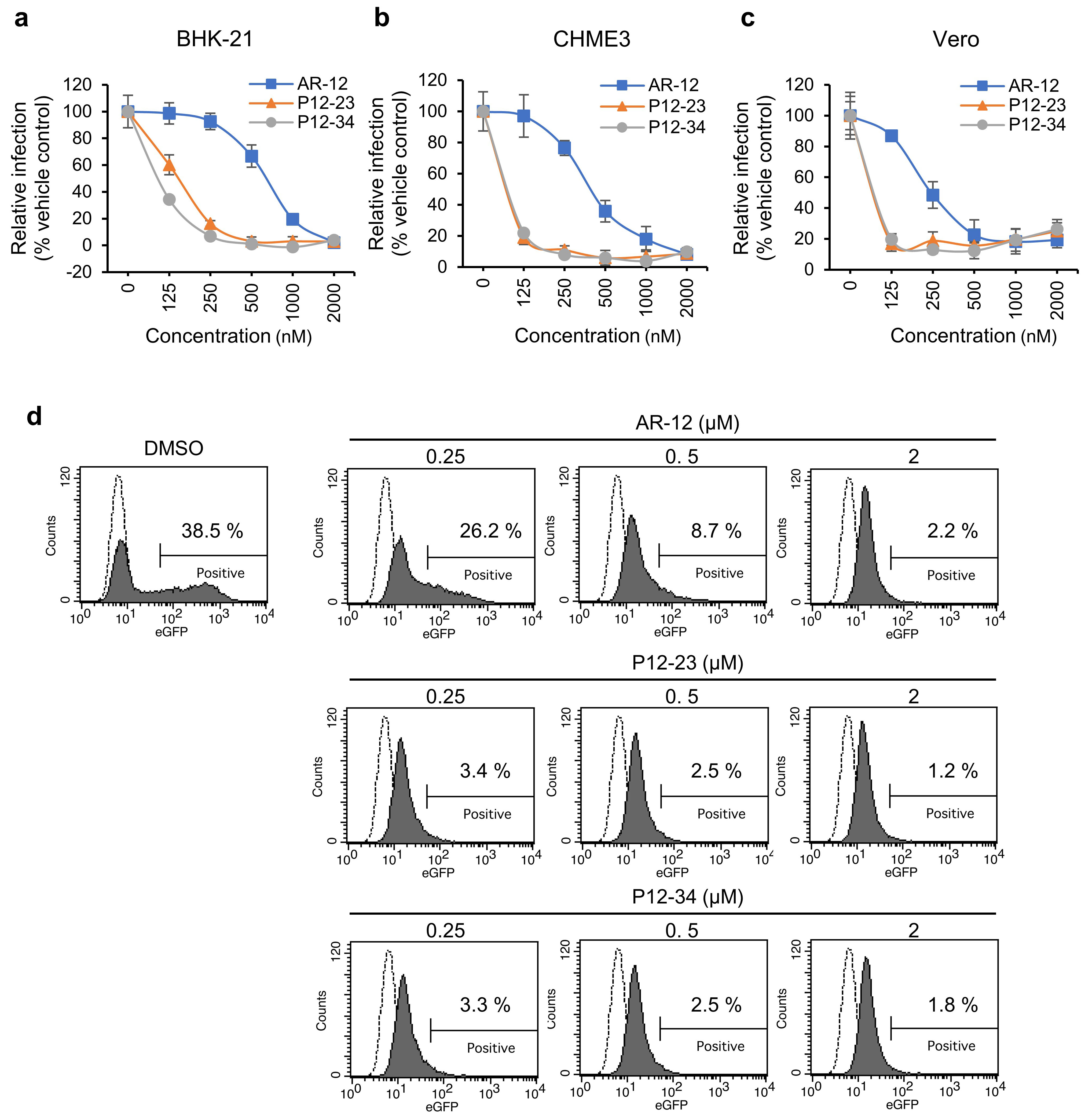

Supplement: Supplementary file 2 — Supplementary figure 1 [file 41426_2018_191_MOESM2_ESM.jpg]

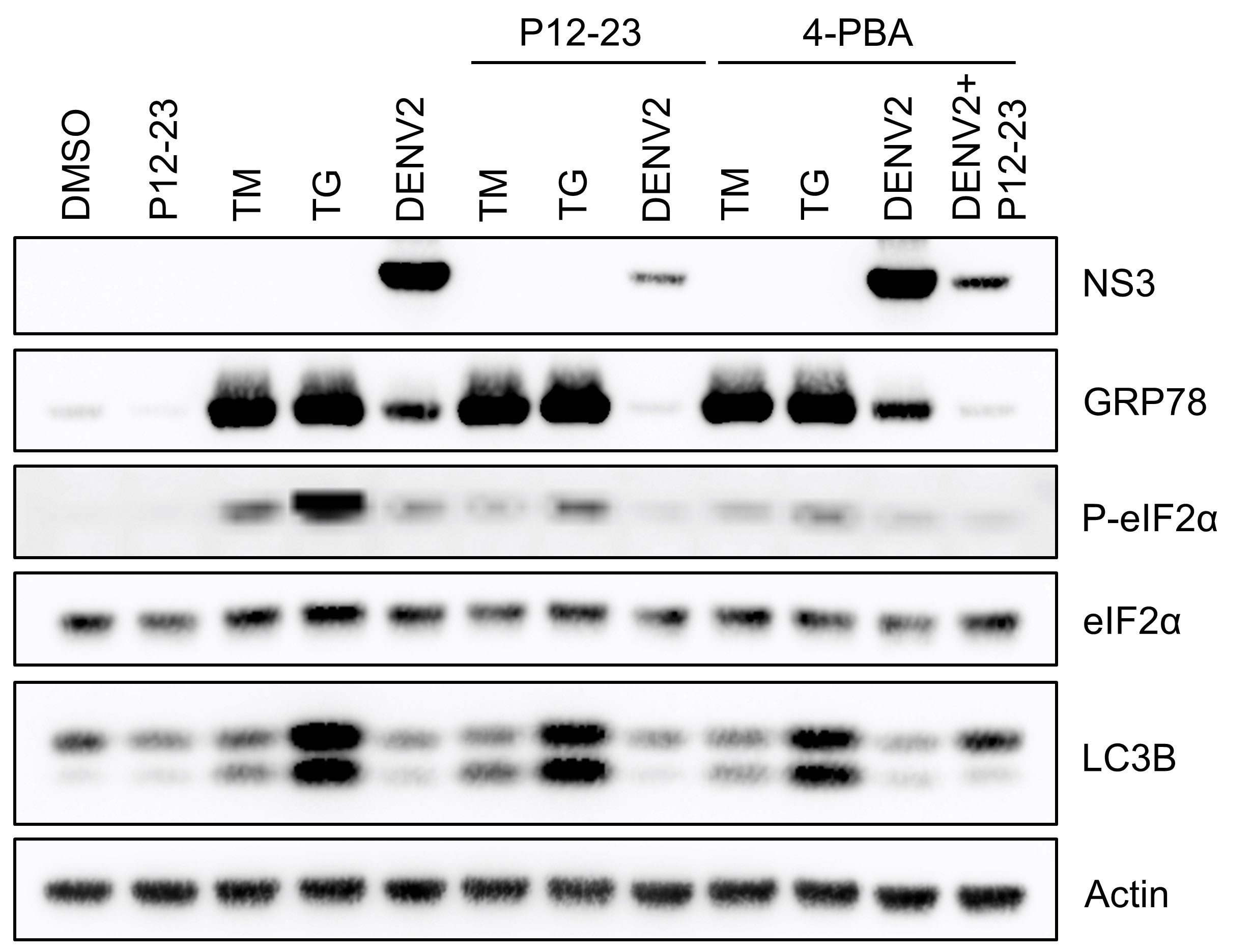

Supplement: Supplementary file 3 — Supplementary figure 2 [file 41426_2018_191_MOESM3_ESM.jpg]

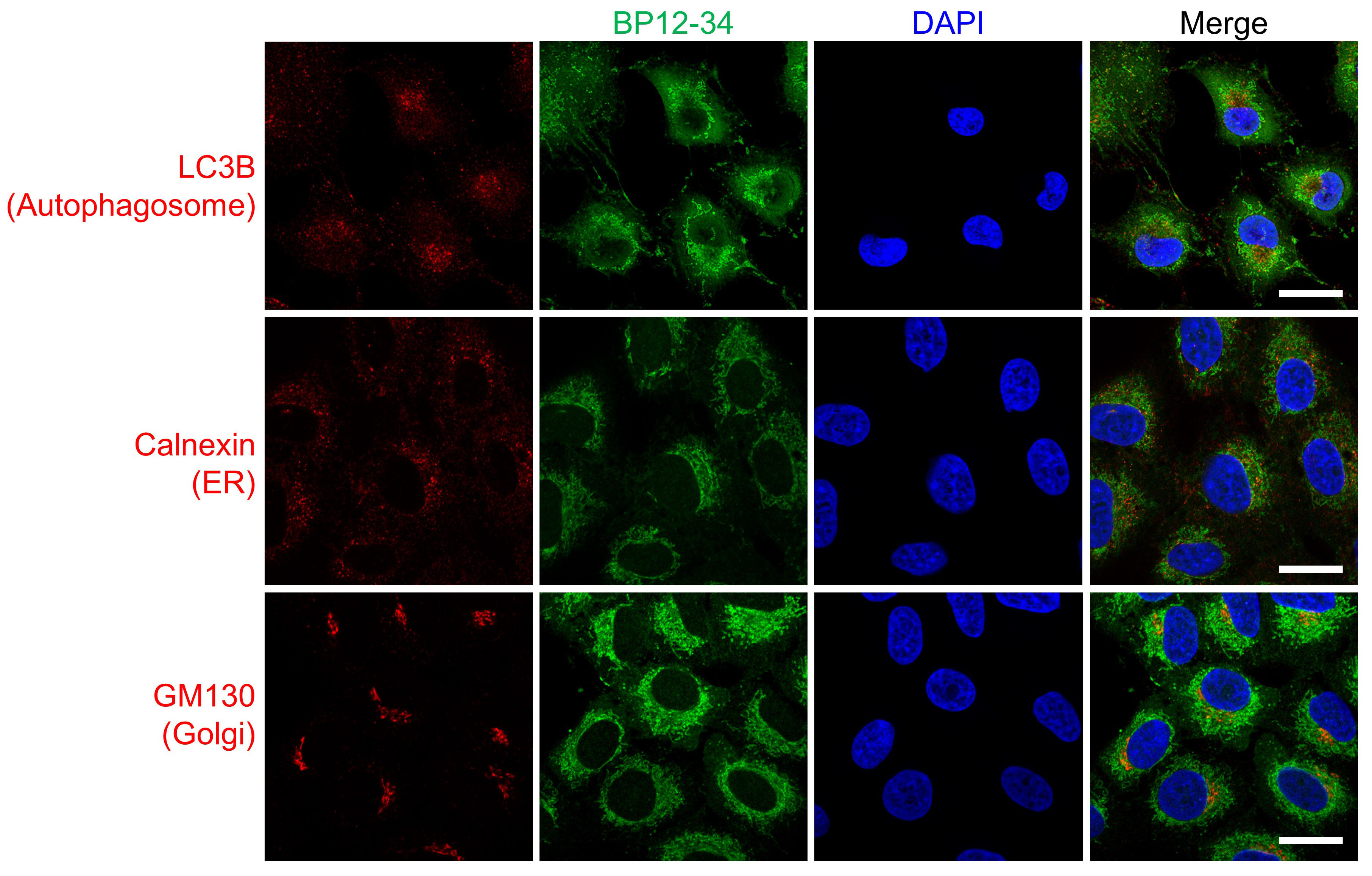

Supplement: Supplementary file 4 — Supplementary figure 3 [file 41426_2018_191_MOESM4_ESM.jpg]

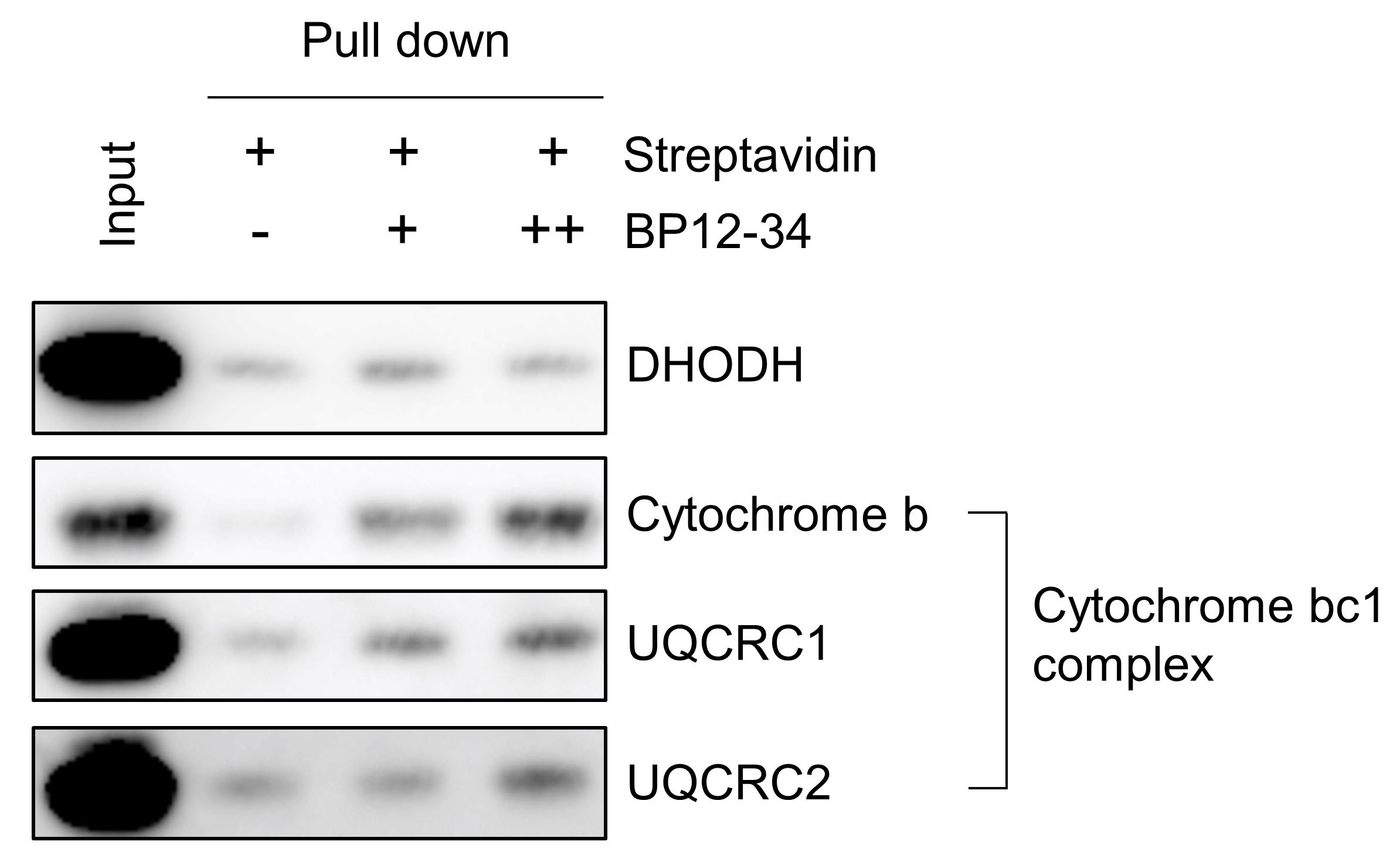

Supplement: Supplementary file 5 — Supplementary figure 4 [file 41426_2018_191_MOESM5_ESM.jpg]

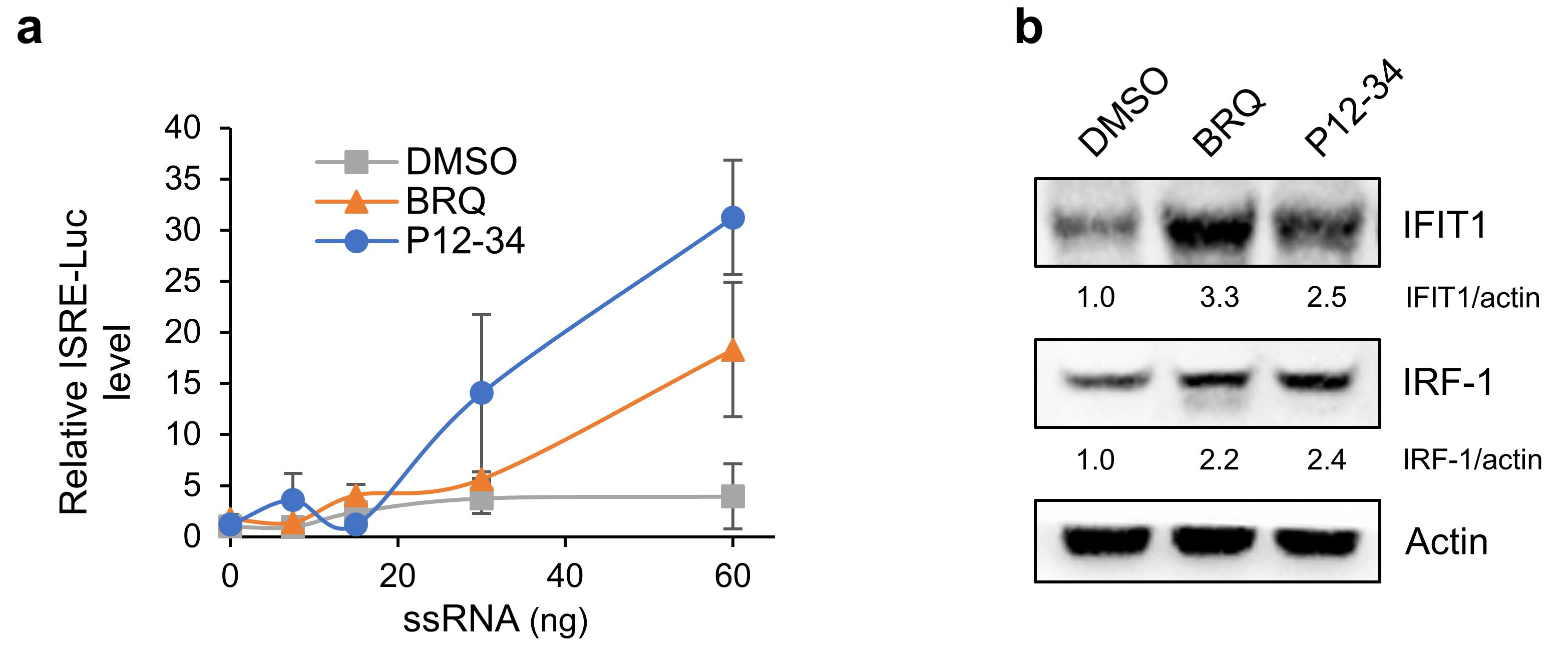

Supplement: Supplementary file 6 — Supplementary figure 5 [file 41426_2018_191_MOESM6_ESM.jpg]
